# Synthetic strategies and therapeutic insights into FDA-approved indole-containing drugs

**DOI:** 10.1080/14756366.2026.2616556

**Published:** 2026-01-27

**Authors:** Tengjiao Yang, Yanfeng Zhang, Peng Liu, Peng Qi, Xiankai Li, Wubin Zhi, Lijie Zhao

**Affiliations:** ^a^ Department of Pharmacy, The Third People’s Hospital of Henan Province, Zhengzhou, Henan, China; ^b^ Shangqiu Municipal Hospital, Shangqiu, Henan, China; ^c^ Institute of Chemistry, Henan Academy of Sciences, Zhengzhou, Henan, China; ^d^ Department of Internal Medicine, The Rogel Cancer Center, University of Michigan, Ann Arbor, MI, USA

**Keywords:** Indole, FDA-approved drugs, synthesis, clinical application

## Abstract

Indole is a privileged heteroaromatic scaffold in medicinal chemistry, characterised by its unique physicochemical properties, hydrogen-bonding potential, and bioisosteric versatility. Over the past decades, numerous indole-containing drugs have been approved by the Food and Drug Administration (FDA), spanning diverse therapeutic areas including oncology, infectious diseases, gastrointestinal disorders, neurological conditions, and cardiovascular diseases. This review provides a comprehensive survey of FDA-approved indole-based drugs, with particular emphasis on those approved from 2013 to the present. Representative synthetic strategies are highlighted to illustrate the versatility of the indole framework in drug design. Furthermore, we systematically discuss each drug’s pharmacology, mechanisms of action, and clinical applications. By integrating synthetic chemistry with clinical applications, this review aims to provide medicinal chemists and drug developers with guidance for leveraging indole scaffolds in next-generation therapeutic discovery and development.

## GRAPHICAL ABSTRACT

**Figure:**
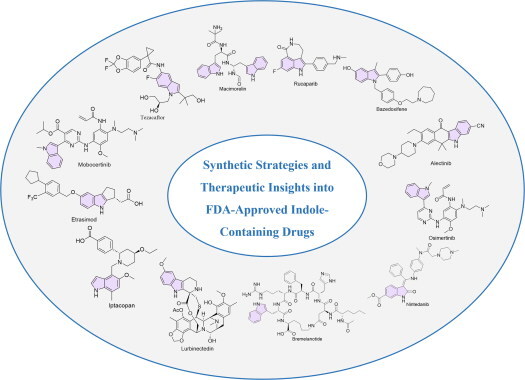

## Introduction

The indole nucleus stands as one of the most versatile and influential heterocyclic scaffolds in medicinal chemistry, owing to its unique structural and electronic features^1^. Its prominence is underscored by its widespread occurrence in natural products and endogenous metabolites, which has long motivated chemists and biologists to exploit its framework for therapeutic design. Indeed, indole and its derivatives are found ubiquitously in nature, spanning plants, fungi, marine organisms, and microorganisms, where they perform diverse biochemical roles, including serving as precursors to alkaloids, signalling molecules, and cofactors^2^.

From a chemical-biological interaction standpoint, the indole core possesses several advantageous properties: its conjugated *π* system supports aromatic stability and favourable overlap with protein binding pockets; the heterocyclic nitrogen enables hydrogen bonding or *π–π* interactions; and its electronic distribution lends itself to functionalization at multiple positions (notably C2, C3, C5–C7), thus providing medicinal chemists with rich opportunities for structural diversification and optimisation^3^. Owing to these features, indole scaffolds have been incorporated into numerous bioactive small molecules spanning a broad therapeutic spectrum, including anticancer, antiviral, anti-inflammatory, neuroprotective, and metabolic disease agents^1^. However, out of the many indole derivatives explored in preclinical settings, only a subset has successfully advanced through clinical development to the Food and Drug Administration (FDA) approval. These approved indole-containing drugs represent especially instructive case studies in how the indole scaffold can be harnessed in practical drug discovery, overcoming challenges of potency, selectivity, pharmacokinetics, toxicity, and resistance.

Despite the wealth of literature on indole derivatives generally, there remains a need for a focused, up-to-date treatment that bridges synthetic methodology and clinical application, especially emphasising those agents that have navigated the stringent regulatory pathway to FDA approval. Previous reviews have catalogued indole-containing pharmaceuticals and explored structure–activity relationships and biological targets^4^. However, fewer works provide an integrated view combining the synthetic routes and clinical application of FDA-approved indole drugs.

**Figure 1.**
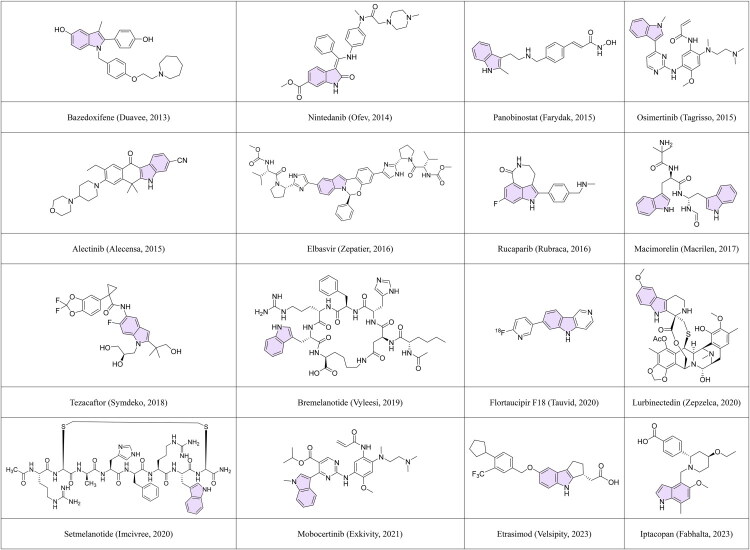
FDA-approved indole-containing drugs.

In this review, we present an overview of FDA-approved indole-containing drugs from 2013 to the present (Table 1 and Figure 1). Then, representative synthetic strategies are described, emphasising reactions and tactics that enable the installation, functionalization, or modification of the indole core. Next, for each drug (or class of drugs), we discuss key mechanism of action and clinical applications. Through this synthetic-to-clinical lens, our goal is to provide medicinal chemists, pharmacologists, and drug developers with insights and guidance on leveraging the indole scaffold in future drug discovery.

**Table 1.** FDA-approved indole-containing drugs.

No.	Drug	Brand name	Company	Approval date	Indication
1	Bazedoxifene	Duavee	Pfizer	2013	Moderate-to-severe vasomotor symptoms (hot flashes) in menopausal women; prevention of postmenopausal osteoporosis
2	Nintedanib	Ofev	Boehringer Ingelheim	2014	Idiopathic pulmonary fibrosis
3	Panobinostat	Farydak	Novartis	2015	Relapsed/refractory multiple myeloma
4	Osimertinib	Tagrisso	AstraZeneca	2015	Epidermal growth factor receptor (EGFR) T790M + metastatic non-small cell lung cancer (NSCLC)
5	Alectinib	Alecensa	Roche/Genentech	2015	Anaplastic lymphoma kinase (ALK) + metastatic NSCLC (later expanded to 1st-line and adjuvant)
6	Elbasvir	Zepatier	Merck	2016	Chronic hepatitis C virus (HCV) genotype 1 or 4
7	Rucaparib	Rubraca	Clovis Oncology	2016	BRCA-mutated ovarian cancer; later maintenance and mCRPC
8	Macimorelin	Macrilen	Aeterna Zentaris	2017	Diagnosis of adult growth hormone deficiency (AGHD)
9	Tezacaftor	Symdeko	Vertex Pharmaceuticals	2018	Cystic fibrosis (≥6 years, specific CFTR mutations)
10	Bremelanotide	Vyleesi	AMAG/Covis Pharma	2019	Acquired, generalised hypoactive sexual desire disorder (HSDD) in premenopausal women
11	Flortaucipir F18	Tauvid	Lilly	2020	PET imaging of tau pathology in Alzheimer’s disease
12	Lurbinectedin	Zepzelca	Jazz Pharmaceuticals	2020	Metastatic small cell lung cancer (SCLC) with disease progression on/after platinum-based chemo
13	Setmelanotide	Symdeko	Rhythm Pharmaceuticals	2020	Obesity
14	Mobocertinib	Exkivity	Takeda	2021	EGFR exon 20 insertion + metastatic NSCLC (accelerated approval)
15	Etrasimod	Velsipity	Pfizer	2023	Moderately to severely active ulcerative colitis
16	Iptacopan	Fabhalta	Novartis	2023	Paroxysmal nocturnal haemoglobinuria

## FDA-approved indole-containing drugs

### Bazedoxifene (Duavee)

Bazedoxifene was approved by the FDA in 2013 as part of the fixed-dose combination conjugated oestrogens/bazedoxifene (Duavee) for treatment of moderate to severe vasomotor symptoms (hot flashes) in postmenopausal women with a uterus, and for the prevention of postmenopausal osteoporosis^5^. Bazedoxifene is a selective oestrogen receptor modulator (SERM) that acts as an oestrogen receptor agonist in bone, and antagonist in breast and uterine tissues. Mechanistically, Bazedoxifene binds to oestrogen receptor (ER) *α* and *β* isoforms, modulating receptor conformation to promote recruitment of coactivators in some tissues and corepressors in others, thereby preserving bone density while limiting oestrogen risks in uterus and breast. In preclinical pharmacodynamics, Bazedoxifene decreased bone resorption markers, reduced osteoclastogenesis, induced osteoclast apoptosis, preserved bone histology, increased bone mineral density in ovariectomized animal models, and showed favourable pharmacokinetics with oral bioavailability (∼6%), high plasma protein binding (∼98–99%), metabolism via glucuronidation, and a half-life supporting once-daily dosing^6^. Bazedoxifene has been evaluated in a Phase IIb study (NCT04821141), which assesses the effect of Bazedoxifene plus conjugated oestrogens on breast imaging and tissue biomarkers in peri- or post-menopausal women at increased risk for developing breast cancer. Serious risks involve venous thromboembolism and, when combined with conjugated oestrogens, an increased risk of endometrial cancer if used without progestins in women with an intact uterus^6^.

**Scheme 1.**
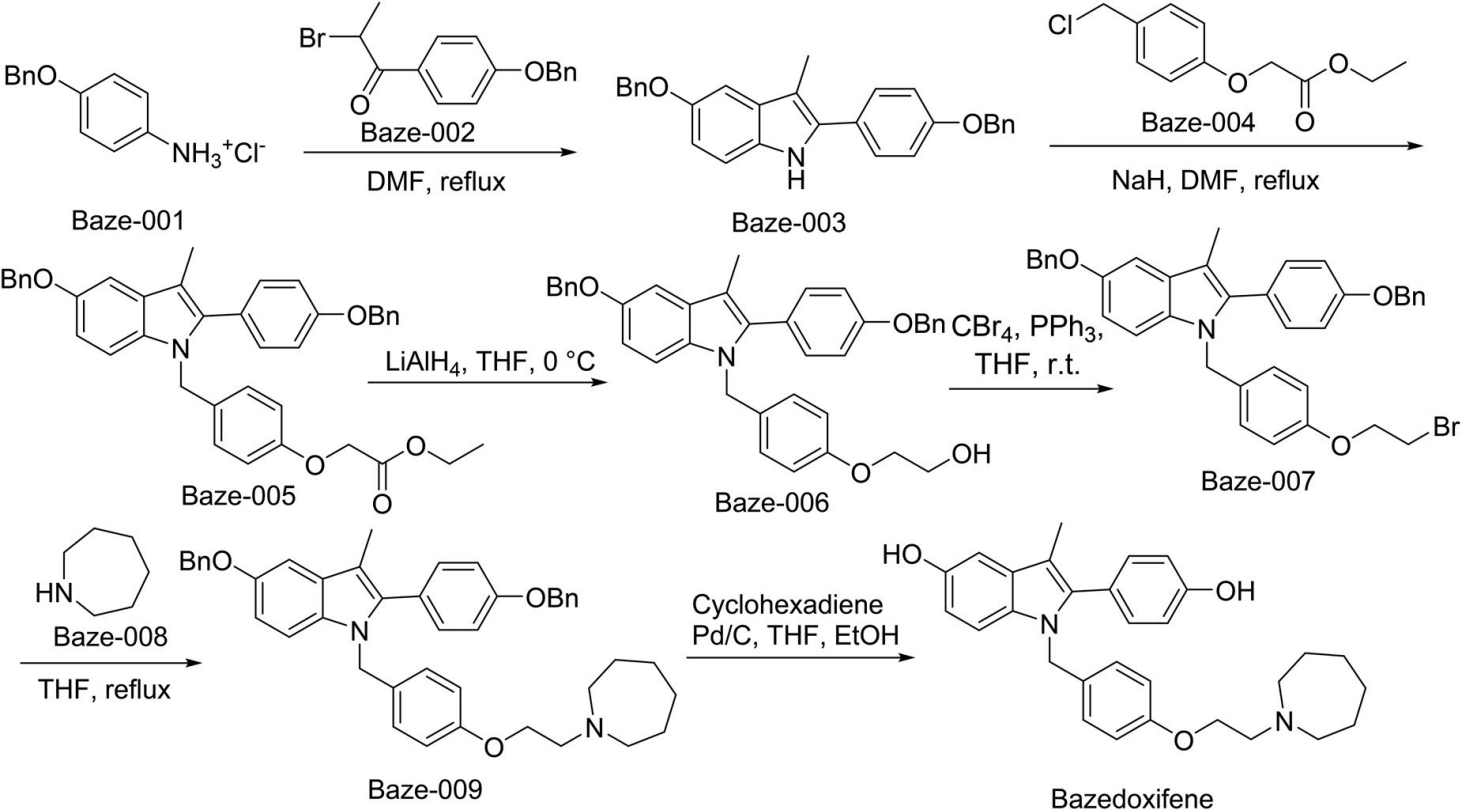
Synthesis of Bazedoxifene.

The synthetic route of Bazedoxifene is illustrated in Scheme 1^7^^,^^8^. The synthesis commences with a regioselective nucleophilic substitution between Baze-001 and Baze-002, affording the key intermediate Baze-003. Subsequent successive nucleophilic displacement of Baze-003 with Baze-004 in an aprotic solvent furnishes Baze-005. This intermediate is then subjected to stereospecific catalytic hydrogenation, yielding Baze-006. Treatment of Baze-006 with a phosphine–halide system induces an Appel transformation, generating the halogenated derivative Baze-007. Under kinetically controlled conditions, Baze-007 undergoes another nucleophilic substitution with Baze-008 to form Baze-009. Final concurrent reduction and hydroxyl protection by catalytic hydrogenation delivers Bazedoxifene with defined stereochemistry.

### Nintedanib (Ofev)

Nintedanib was approved by the FDA in 2014 for the treatment of idiopathic pulmonary fibrosis (IPF) in adults^9–11^. Subsequently, in 2020, it also received FDA approval for treatment of chronic fibrosing interstitial lung diseases (ILDs) with a progressive phenotype, including systemic sclerosis-associated ILD (SSc-ILD). Nintedanib is a small-molecule tyrosine kinase inhibitor (TKI) that targets multiple receptor and non-receptor kinases, particularly platelet-derived growth factor receptor (PDGFR) *α*/*β*, fibroblast growth factor receptors (FGFR) 1–3, vascular endothelial growth factor receptors (VEGFR) 1–3, Fms-like tyrosine kinase 3 (FLT-3), and non-receptor kinases^12^. Mechanistically, by inhibiting the ATP binding site, Nintedanib suppresses fibroblast proliferation, migration, and transformation, reduces angiogenesis, and thereby slows lung fibrosis progression^9^. Preclinical pharmacodynamics demonstrated potent inhibition of kinase autophosphorylation, blockade of fibroblast activation and proliferation in lung-fibrosis animal models, reduction of collagen deposition, and favourable pharmacokinetic profile supporting twice-daily oral dosing. The INBUILD trial (NCT02999178) assessed its efficacy in progressive fibrosing interstitial lung diseases. Additionally, the INPULSIS-1 and INPULSIS-2 trials focused on idiopathic pulmonary fibrosis^13^. Regarding toxicity, the most common adverse events include gastrointestinal effects, elevated liver enzymes, weight loss, and decreased appetite; serious risks include increased bleeding risk, hepatic injury, and potential embryo-foetal toxicity.

**Scheme 2.**
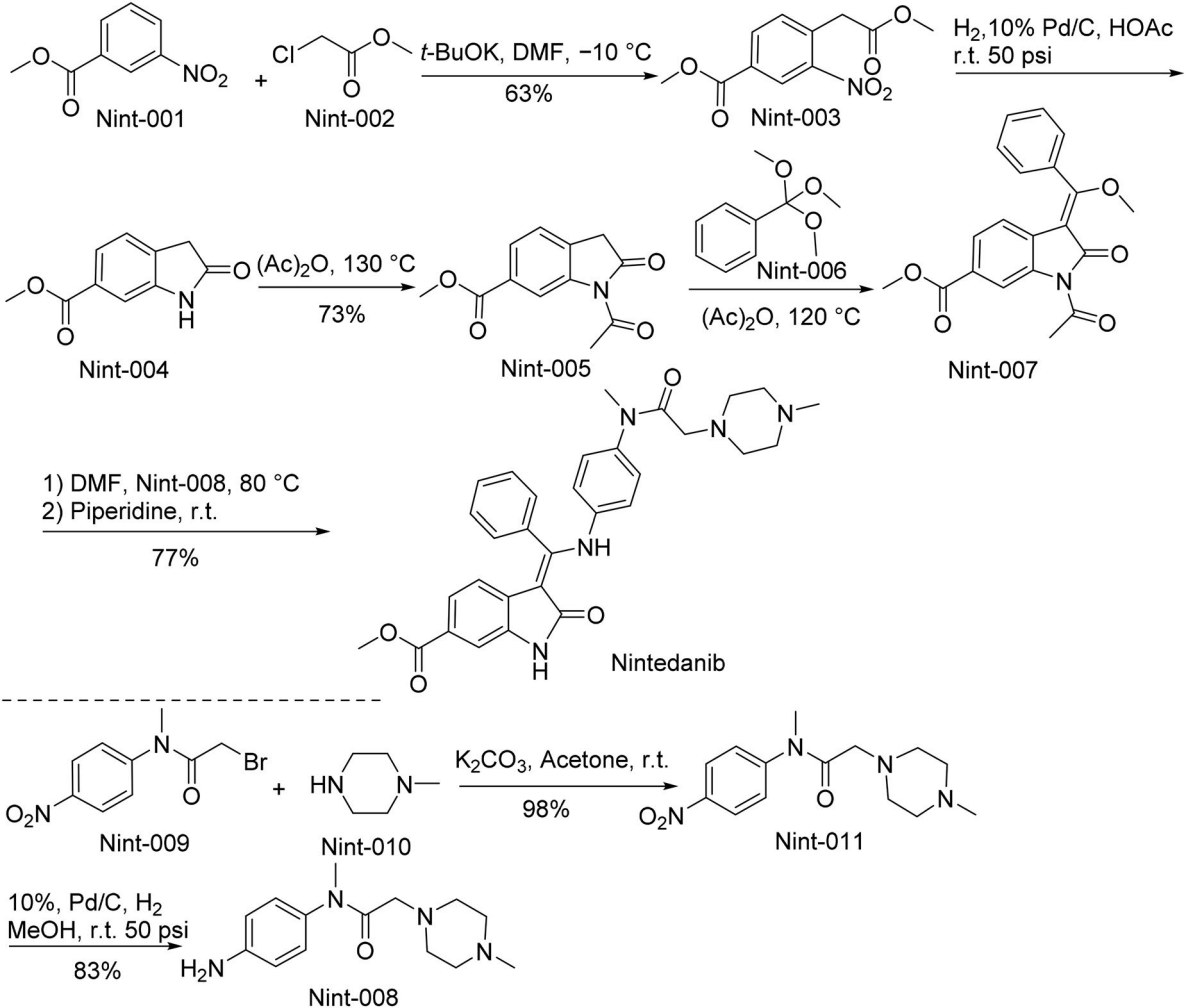
Synthesis of Nintedanib.

The synthetic route of Nintedanib is shown in Scheme 2^14^. Nint-001 reacts with Nint-002 through ‌amine-directed aromatic nucleophilic substitution‌ to form aryl ether Nint-003‌, which undergoes ‌nitro group reduction coupled with amide formation‌ to yield Nint-004‌. Subsequent ‌Schotten-Baumann acylation‌ with acetic anhydride produces acetyl-protected Nint-005‌, followed by ‌amide condensation‌ with Nint-006 to generate Nint-007‌. Final ‌halide substitution‌ between Nint-007 and Nint-008 yields Nintedanib. Concurrently, Nint-009 undergoes ‌alkylation‌ with Nint-010 via nucleophilic substitution to form benzyl ether Nint-011‌, which is ‌catalytically reduced‌ to produce alcohol Nint-012‌.‌

### Panobinostat (Farydak)

Panobinostat was approved by the FDA in 2015 under accelerated approval as part of a combination therapy with bortezomib and dexamethasone for the treatment of relapsed or refractory multiple myeloma in patients who had received at least two prior regimens, including bortezomib and an immunomodulatory agent^15^. It is a potent, pan–histone deacetylase (HDAC) inhibitor, inhibiting multiple classes of HDAC enzymes (including class I, II, and IV), leading to accumulation of acetylated histones and non-histone proteins that modulate gene expression, cell cycle arrest, differentiation, and apoptosis in malignant cells^16^. Mechanistically, by inhibiting HDAC activity, Panobinostat increases transcription of tumour suppressor genes, alters chromatin structure, disrupts DNA repair, triggers unfolded protein response, and enhances proteasome inhibitor activity in multiple myeloma cells. In preclinical pharmacodynamics, Panobinostat demonstrated cytotoxic activity against a broad range of tumour cell lines and xenograft models, including multiple myeloma and solid tumours. It induced apoptosis, inhibited proliferation, and sensitised cells to other anticancer agents^17^. The efficacy of Panobinostat was evaluated in a randomised, double-blind, Phase III study (NCT01023308), which demonstrated that the addition of Panobinostat to Bortezomib and Dexamethasone significantly improved progression-free survival in patients with relapsed or relapsed and refractory multiple myeloma^18^. Common adverse events associated with Panobinostat include thrombocytopenia, leukopoenia, anaemia, diarrhoea, nausea, vomiting, fatigue, and peripheral edoema. Serious adverse events may involve severe diarrhoea and cardiovascular issues such as cardiac ischaemia and arrhythmias, including QTc interval prolongation^19^.

**Scheme 3.**
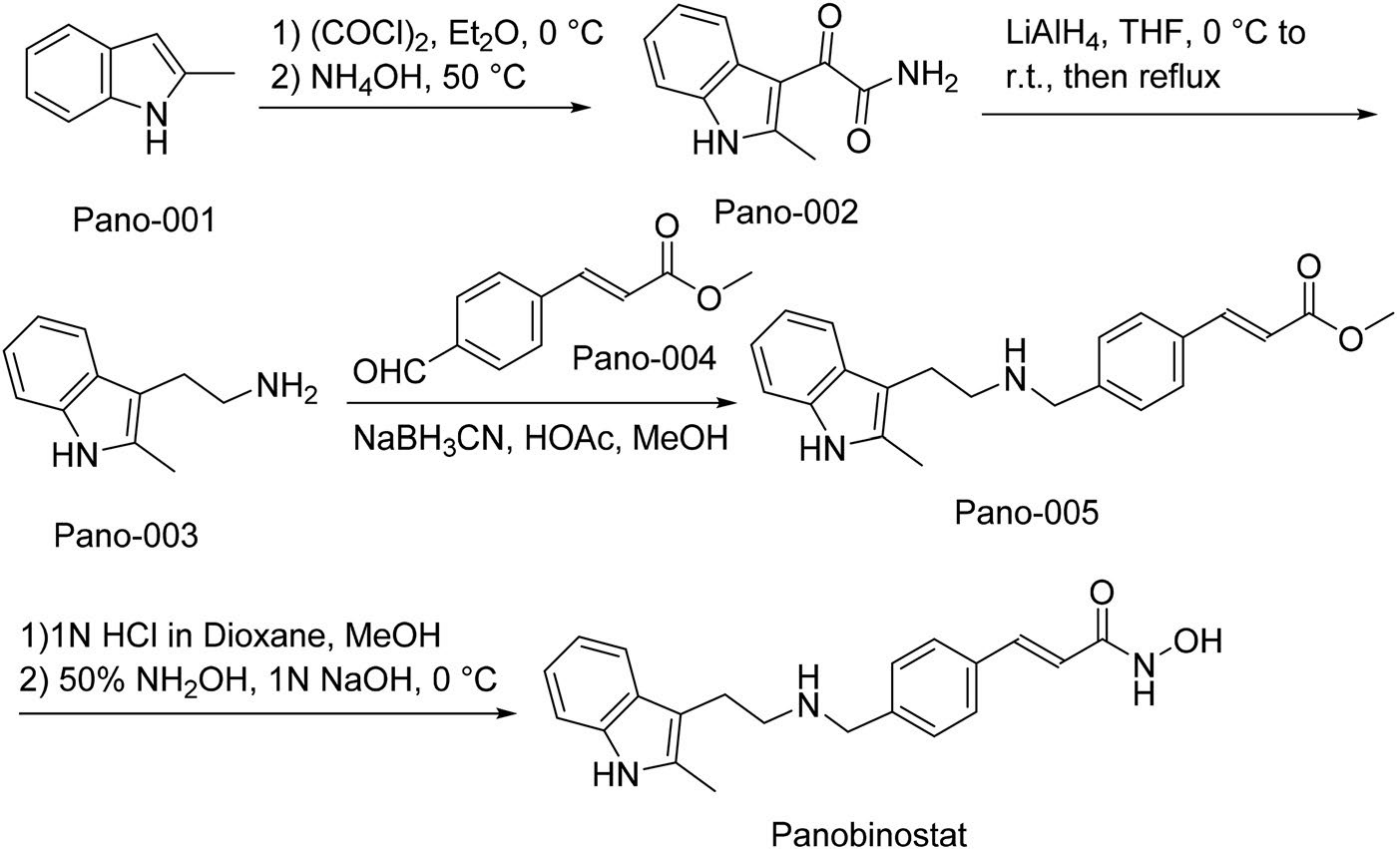
Synthesis of Panobinostat.

The synthetic route of Panobinostat is shown in Scheme 3^20^^,^^21^. The synthesis commences with the conversion of Pano-001 to its corresponding acyl chloride intermediate through oxalyl chloride-mediated activation under strictly anhydrous conditions‌. Subsequent nucleophilic ammonolysis of the reactive acyl chloride species yields carboxamide derivative Pano-002‌. Catalytic hydrogenation of Pano-002 under controlled hydrogen pressure achieves selective nitro group reduction, generating amine intermediate Pano-003‌. Pano-003 then undergoes catalytic reductive amination with carbonyl-containing Pano-004 in the presence of sodium triacetoxyborohydride, forming the critical C-N bond in Pano-005‌. The terminal synthesis stage involves sequential transformations: base-mediated ester hydrolysis of Pano-005 liberates the carboxylic acid functionality, followed by carbodiimide-activated amidation with the target amine partner to assemble the final hydroxamic acid pharmacophore of Panobinostat‌.

### Osimertinib (Tagrisso)

Osimertinib received FDA approval in 2015 for the treatment of metastatic NSCLC harbouring the EGFR T790M resistance mutation following disease progression on prior EGFR TKI therapy^22–24^. It later gained additional approvals: in 2018 as a first-line option for patients with EGFR exon 19 deletions or L858R mutations, and in 2020 as adjuvant therapy for completely resected, EGFR-mutant NSCLC. As a third-generation, irreversible EGFR TKI, Osimertinib exhibits selective inhibition of both classical activating EGFR alterations (e.g. exon 19 deletions and L858R) and the T790M gatekeeper mutation, while sparing wild-type EGFR to a greater extent than earlier agents. Its mechanism involves covalent modification of Cys797 within the ATP-binding pocket of mutant EGFR, thereby suppressing downstream oncogenic signalling cascades—particularly the MAPK and PI3K/AKT pathways—that promote tumour cell survival and proliferation^25^. Preclinical studies demonstrated that Osimertinib potently suppresses EGFR phosphorylation in cell lines expressing activating and T790M mutations, induces tumour regression in xenograft models, and achieves enhanced central nervous system penetration compared to first- and second-generation EGFR inhibitors^26^. The pivotal FLAURA trial (NCT02296125), a Phase III study in treatment-naïve patients with EGFR-mutated advanced NSCLC, showed that Osimertinib significantly prolonged progression-free survival relative to standard first-generation TKIs (Gefitinib or Erlotinib). An ongoing Phase III trial (NCT04035486) is currently evaluating the combination of Osimertinib with platinum-based chemotherapy as initial therapy in this population, with the goal of determining whether dual-modality treatment improves clinical outcomes over Osimertinib monotherapy. Common side effects of Osimertinib include diarrhoea, skin rash, xerosis, and nail changes. More serious, though less frequent, adverse events may involve interstitial lung disease, QTc interval prolongation, and cardiac dysfunction such as cardiomyopathy.

**Scheme 4.**
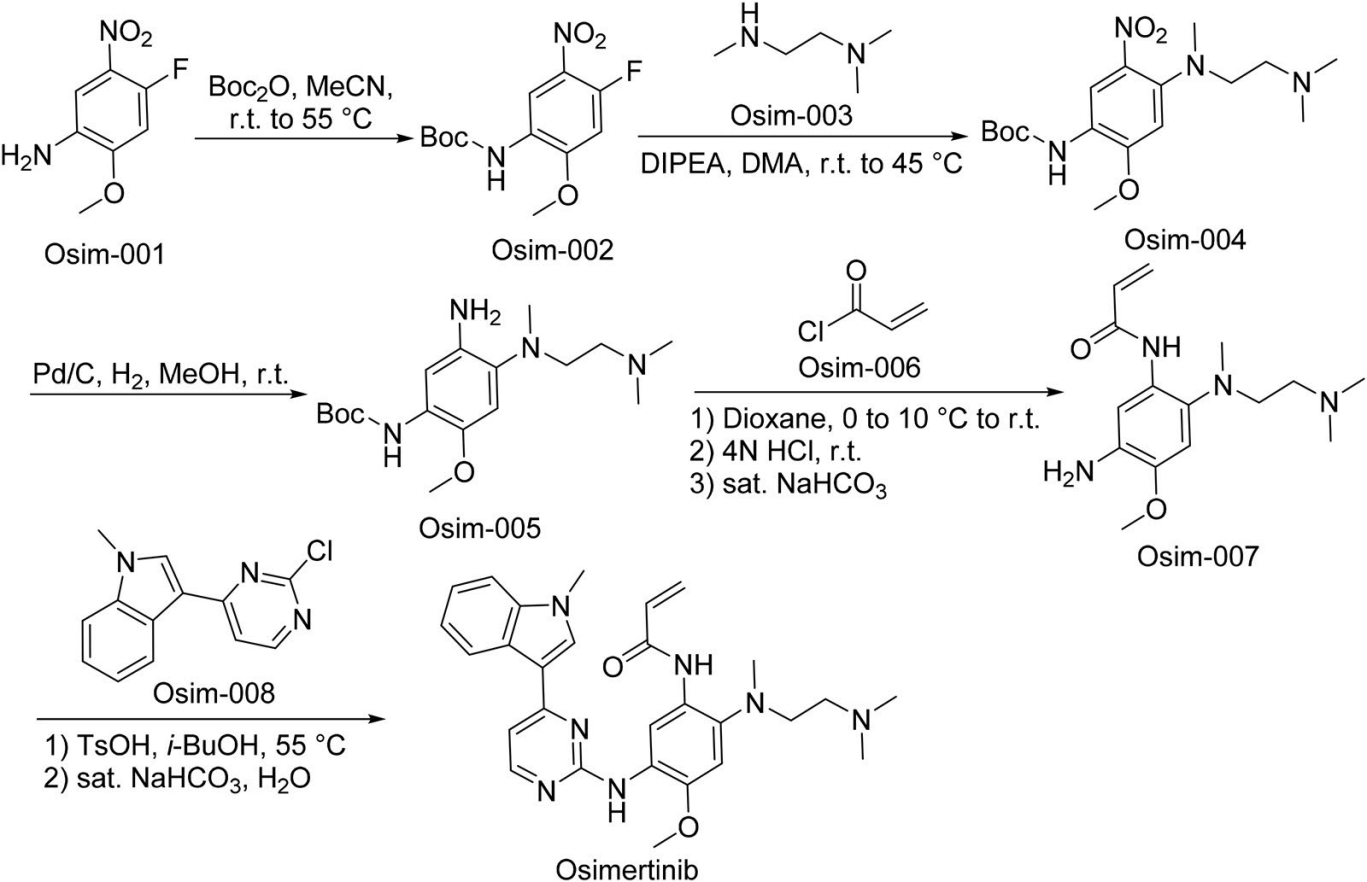
Synthesis of Osimertinib.

The synthetic route of Osimertinib is shown in Scheme 4^27^. The synthesis initiates with ‌regioselective SNAr displacement‌ of Osim-001 to install a pyridinyl sulphonamide moiety, yielding Osim-002‌. Subsequent ‌Buchwald-Hartwig coupling‌ between Osim-002 and aryl chloride Osim-003 generates biaryl ether intermediate Osim-004‌.‌ Catalytic hydrogenation‌ of Osim-004 selectively reduces the nitro group to an amine, producing Osim-005 while preserving the aromatic system‌. This intermediate undergoes ‌HATU-mediated amidation‌ with carboxylic acid Osim-006 to form carboxamide derivative Osim-007‌. The synthesis concludes *via* ‌stereospecific nucleophilic substitution‌ between Osim-007 and chiral epoxide Osim-008, producing Osimeritinib.

### Alectinib (Alecensa)

Alectinib was first approved by the FDA in 2015 for the treatment of patients with anaplastic lymphoma kinase (ALK)-positive metastatic NSCLC who had progressed on or were intolerant to crizotinib^28^. In 2017, FDA granted full approval for the use of Alectinib as first-line therapy in metastatic ALK-positive NSCLC. In 2024, FDA approved Alectinib as adjuvant treatment following tumour resection in stage IB-IIIA ALK-positive NSCLC^29^. Alectinib is a highly selective small-molecule inhibitor of ALK, including ALK fusion proteins and many resistance mutations, with active metabolite M4 contributing similarly. Mechanistically, it binds the ATP-binding domain of ALK, preventing downstream signalling via pathways such as STAT3, PI3K/AKT, MAPK/ERK, causing suppression of proliferation and induction of apoptosis in ALK-driven tumours. In preclinical pharmacodynamics, Alectinib demonstrated potent inhibition of ALK phosphorylation in ALK-rearranged cell lines, suppressed growth in xenograft models, including brain metastasis models, showed favourable pharmacokinetics with good brain penetration and a long half-life supporting twice daily dosing^29^^,^^30^. Clinical efficacy: in the pivotal ALEX trial comparing Alectinib *versus* crizotinib in treatment-naïve patients, Alectinib significantly improved progression-free survival (median PFS ∼34.8 *vs.* ∼10.9 months), reduced CNS progression, and had higher intracranial response rates^29^. In earlier crizotinib-refractory setting (per accelerated approval), response rates in single-arm studies were ∼50–60% with durable responses^31^. Regarding toxicity, Alectinib is generally well tolerated; common adverse events include constipation, fatigue, peripheral edoema, myalgia, increased liver enzymes, and bradycardia^32^. Serious adverse effects include interstitial lung disease/pneumonitis, hepatotoxicity, heart rate abnormalities (including sinus bradycardia), and embryo-foetal toxicity in animal studies^32^.

**Scheme 5.**
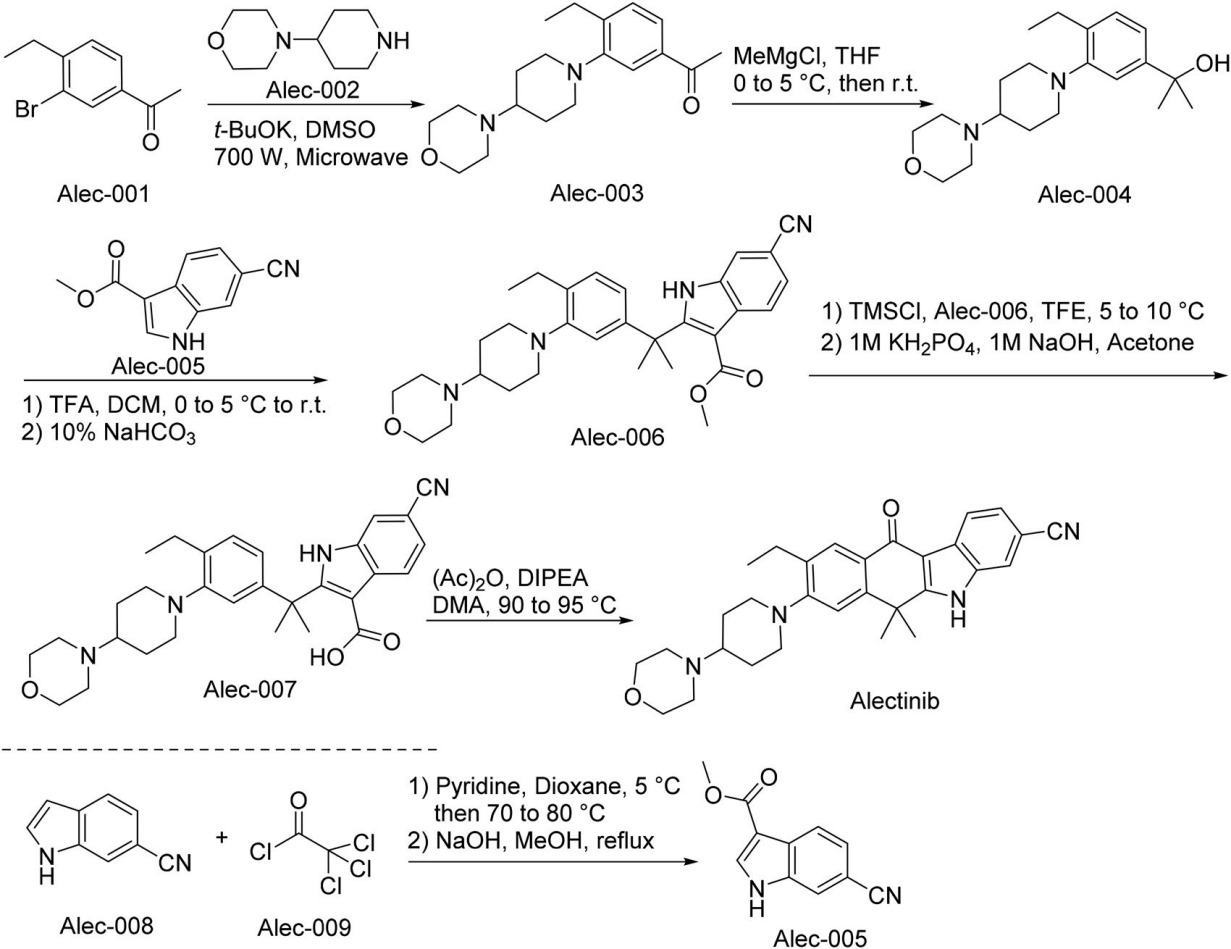
Synthesis of Alectinib.

The synthetic route of Alectinib is shown in Scheme 5^33^. Alec-001 undergoes ‌SNAr displacement‌ with Alec-002 to yield aryl ether intermediate Alec-003 under anhydrous conditions‌. Alec-003 subsequently participates in a ‌Grignard reagent addition‌ at low temperature, forming tertiary alcohol Alec-004 through stereoselective carbonyl attack‌. Alec-004 engages in an ‌AlCl_3_-catalyzed Friedel-Crafts alkylation‌ with Alec-005, yielding Alec-006‌. Acidic hydrolysis of the ester moiety in Alec-006 generates carboxylic acid derivative Alec-007‌. A final ‌intramolecular Friedel-Crafts cyclization‌ of Alec-007 yields Alectinib.

Parallel Synthesis of Alec-005‌: Alec-008 and Alec-009 undergo ‌SN_2_ displacement‌ in polar aprotic solvent, producing benzyl chloride intermediate Alec-005 with inversion of configuration.

### Elbasvir (Zepatier)

Elbasvir, in fixed‐dose combination with Grazoprevir (Zepatier), received its first FDA approval in 2016 for the treatment of chronic hepatitis C virus (HCV) genotype 1 or 4 infection in adults, with or without ribavirin, depending on patient characteristics^34^. As an NS5A inhibitor, Elbasvir exerts potent inhibition of the HCV replicon replication complex, required for viral RNA replication and virion assembly; Grazoprevir complements this by inhibiting the NS3/4A protease^35^. Mechanistically, Elbasvir binds to NS5A, reducing both RNA replication and assembly of HCV particles, thereby suppressing viral load. In preclinical pharmacodynamics, Elbasvir exhibited broad genotype activity with low picomolar EC_50_ values, high potency against genotypes 1a, 1b, and 4, and favourable pharmacokinetic properties including high plasma protein binding (>99.9%) and a half-life sustaining once-daily dosing with steady state achieved in ∼6 days^35^. The efficacy of Elbasvir, in combination with Grazoprevir, was evaluated in a Phase III study (NCT02105688), which demonstrated high sustained virologic response rates at 12 weeks (SVR12), indicating the regimen’s effectiveness in this population. Elbasvir, in combination with Grazoprevir, has been evaluated in another Phase III clinical trial (NCT02332707), which assessed the safety and efficacy of an 8-week regimen of Grazoprevir, Ruzasvir, and Uprifosbuvir in patients with hepatitis C virus infection^36^. Common adverse effects of the Elbasvir and Grazoprevir combination include fatigue, headache, nausea, and elevated liver enzymes. Serious adverse events are rare but may involve significant elevations in liver transaminases.

**Scheme 6.**
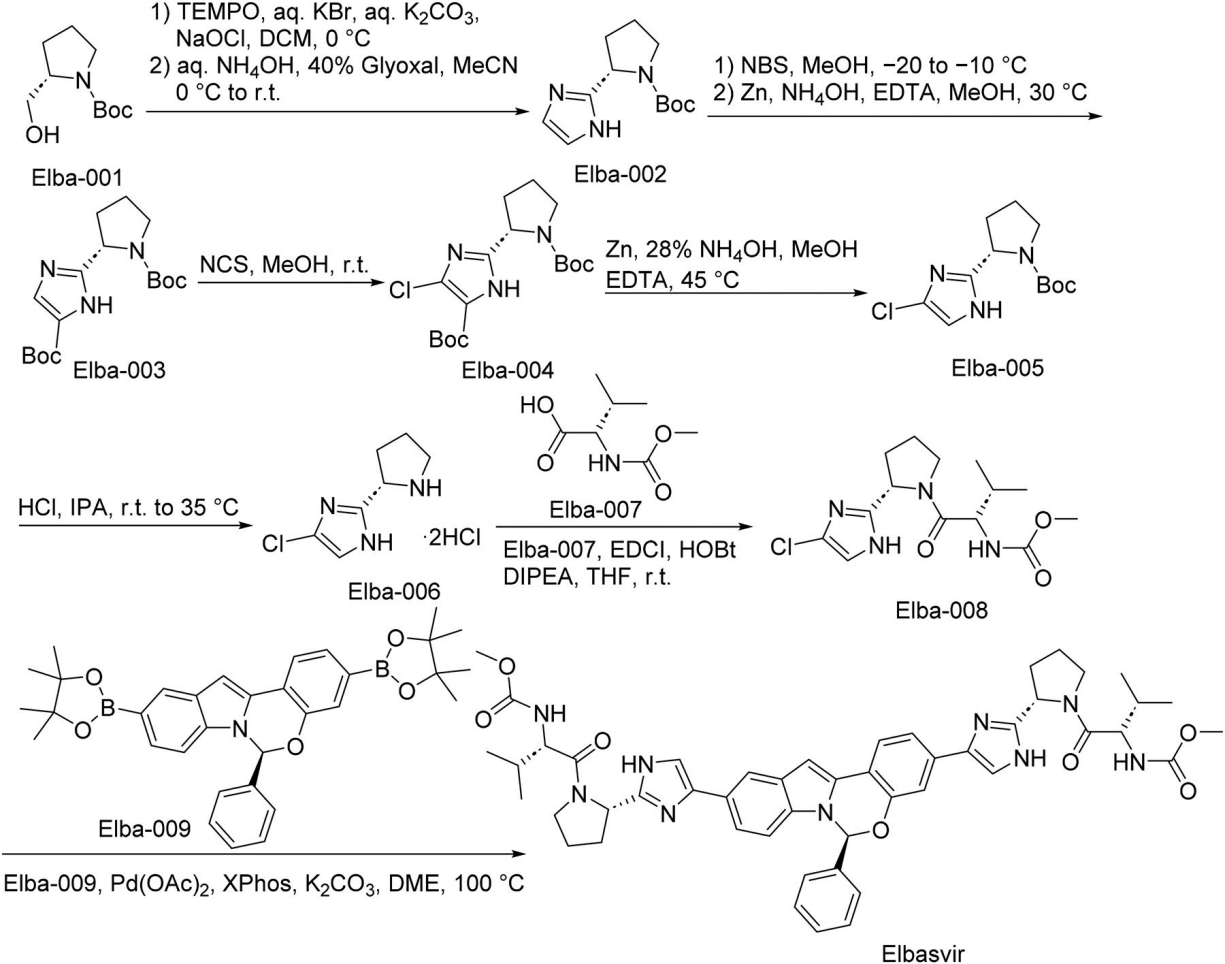
Synthesis of Elbasvir.

The synthetic route of Elbasvir is shown in Scheme 6^37^. Elba-001 is oxidised and cyclized in one pot to afford Elba-002. A protecting-group exchange then furnishes Elba-003, which undergoes chlorination to give Elba-004. Sequential deprotections—first under standard conditions and then with acid—deliver Elba-005 and Elba-006, respectively. Amide coupling of Elba-006 with Elba-007 yields Elba-008, which participates in a Suzuki–Miyaura cross-coupling with Elba-009 to afford Elbasvir.

### Rucaparib (Rubraca)

Rucaparib was first approved by the FDA in 2016 under accelerated approval for the treatment of patients with deleterious cc-mutation (germline and/or somatic)-associated advanced ovarian cancer who have been treated with two or more chemotherapies^38^. It was subsequently approved in 2018 for maintenance treatment of recurrent epithelial ovarian, fallopian tube, or primary peritoneal cancer in adults who achieved complete or partial response to platinum-based chemotherapy. Further expanded in 2020, the FDA granted accelerated approval for use in metastatic castration-resistant prostate cancer (mCRPC) with BRCA mutations after progression on androgen receptor-directed therapy and taxanes. Rucaparib is an oral, small-molecule poly(ADP-ribose) polymerase (PARP) inhibitor that targets PARP1, PARP2, and PARP3 enzymes, exploiting homologous recombination repair deficiency in breast cancer (BRCA)-mutant tumours^39^. Mechanistically, inhibition of PARP leads to accumulation of DNA single-strand breaks that convert to double-strand breaks; in HR-deficient cells, this synthetic lethality induces tumour cell death. In preclinical pharmacodynamics, rucaparib showed potent inhibition of PARP enzymatic activity, demonstrated dose-dependent antitumor activity in BRCA-deficient cell lines and xenograft models, with favourable pharmacokinetics supporting twice-daily dosing^39–41^. The efficacy of rucaparib was evaluated in the TRITON3 trial (NCT02975934), a Phase III study comparing rucaparib to physician’s choice of therapy in patients with metastatic castration-resistant prostate cancer associated with homologous recombination gene deficiency. The trial demonstrated a significant improvement in progression-free survival for patients treated with rucaparib. Common adverse events associated with rucaparib include fatigue, nausea, vomiting, anaemia, and elevations in liver enzymes. Serious adverse events may involve myelosuppression and potential liver toxicity^42^.

**Scheme 7.**
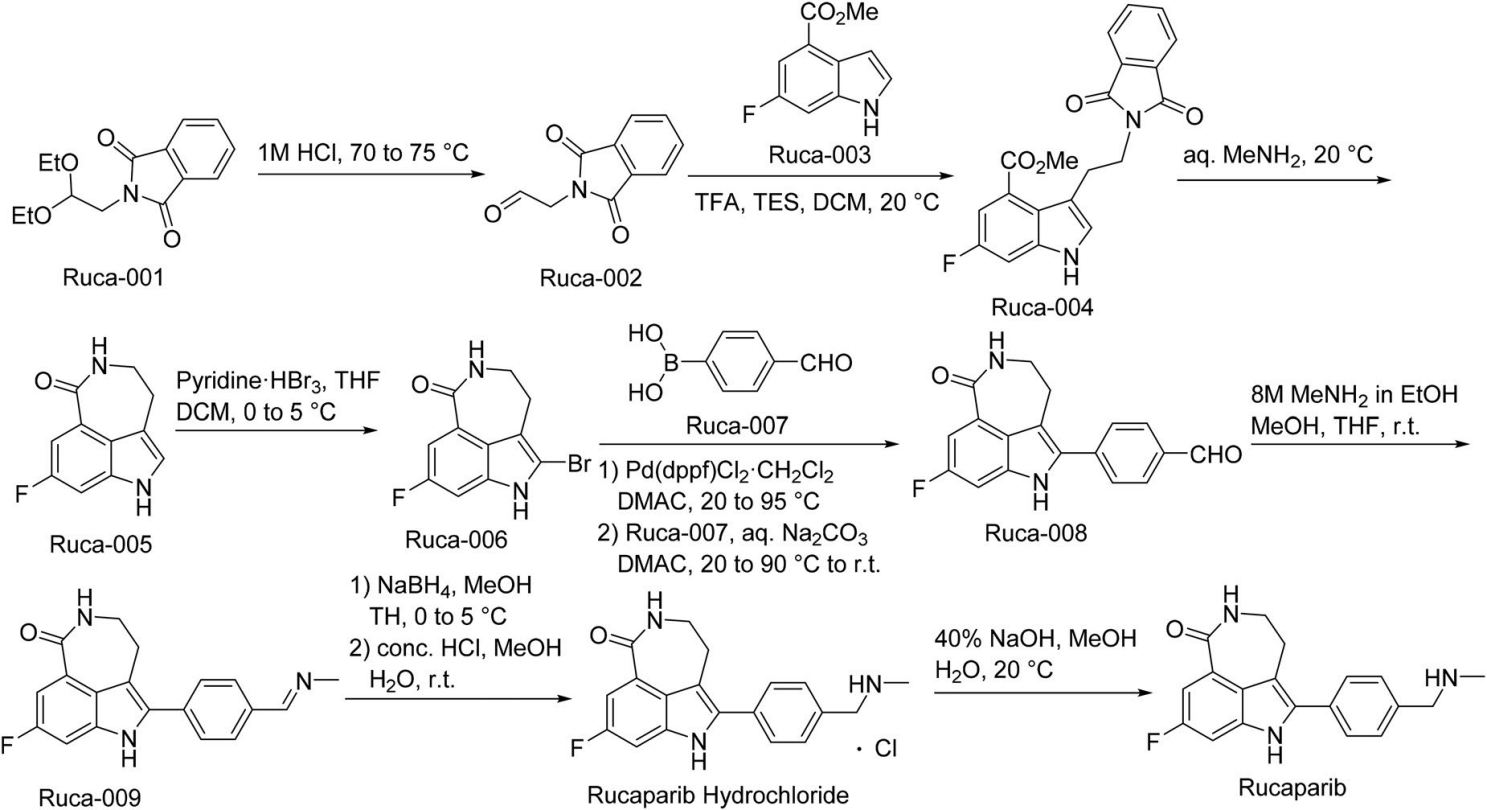
Synthesis of Rucaparib.

The synthetic route of Rucaparib is shown in Scheme 7^43^. The synthesis initiates with acid-catalyzed hydrolysis of Ruca-001 under controlled temperature conditions to yield carboxylic acid intermediate Ruca-002‌. Subsequent nucleophilic displacement of Ruca-002 with Ruca-003 in aprotic solvent generates alkylated product Ruca-004‌. Intramolecular amide cyclisation of Ruca-004 under dehydrating conditions forms the core heterocyclic structure Ruca-005‌. Electrophilic bromination of Ruca-005 at the activated aromatic position produces bromide Ruca-006‌. Palladium-catalyzed Suzuki-Miyaura coupling between Ruca-006 and boronic acid derivative Ruca-007 constructs the biaryl framework in Ruca-008‌. Reductive amination of Ruca-008 with methylamine under hydrogenation conditions introduces the tertiary amine moiety, yielding Ruca-009‌. Final purification involves hydrochloric acid-mediated salt formation to generate Rucaparib Hydrochloride, followed by pH-controlled neutralisation to isolate the free base Rucaparib‌.

### Macimorelin (Macrilen)

Macimorelin, marketed under the brand name Macrilen, is a synthetic growth hormone secretagogue receptor (GHSR) agonist developed by Aeterna Zentaris^44^. The FDA approved Macrilen in 2017 for the diagnosis of adult growth hormone deficiency (AGHD). Macimorelin functions by binding to the GHSR in the pituitary gland and hypothalamus, mimicking the action of ghrelin. This binding stimulates the secretion of growth hormone (GH), facilitating the assessment of GH reserve in diagnostic testing. In preclinical studies, Macimorelin demonstrated potent GH-releasing activity by activating GHSR, leading to significant increases in circulating GH levels. This effect was observed to be dose-dependent and consistent across various animal models. The efficacy of Macimorelin was evaluated in a pivotal Phase III trial (NCT02558829), which assessed its performance as a diagnostic tool for AGHD. The study concluded that Macimorelin is a safe and effective alternative to the insulin tolerance test (ITT) for diagnosing AGHD^45^. Macimorelin has been evaluated in a Phase III study known as the DETECT trial (NCT04786873). This international, multicentre, open-label trial assessed the efficacy and safety of a single oral dose of 1.0 mg/kg Macimorelin acetate as a growth hormone stimulation test in paediatric patients aged 3–17 years with suspected growth hormone deficiency (GHD). The trial enrolled 102 subjects across various countries, including the USA, Germany, and Italy. Top-line results indicated that while macimorelin demonstrated its capacity to stimulate growth hormone release, the primary efficacy endpoint was not met, potentially due to a high false-positive rate in comparator tests. Common adverse effects reported with Macimorelin include dysgeusia, dizziness, headache, fatigue, nausea, hunger, diarrhoea, and upper respiratory tract infections.

**Scheme 8.**
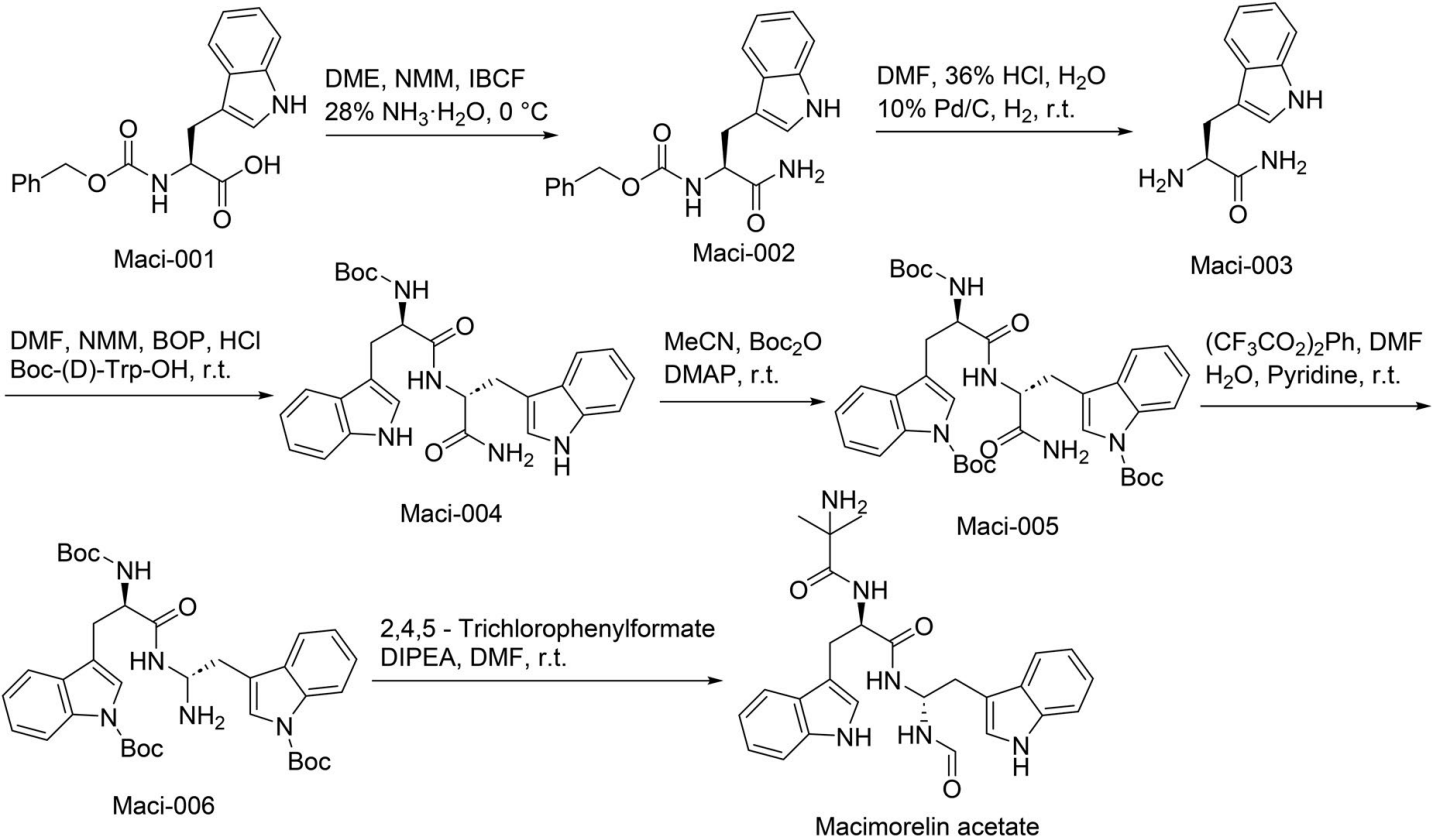
Synthesis of Macimorelin.

The synthetic route of Macimorelin is shown in Scheme 8^46^. The synthetic sequence commences with ammonolytic cleavage of Maci-001 under controlled pH conditions to yield amine intermediate Maci-002‌. Subsequent acid-mediated deprotection of Maci-002 generates free hydroxyl-containing compound Maci-003‌. This intermediate undergoes carbodiimide-activated condensation with a carboxylate partner, forming peptide-linked Maci-004‌. Selective protection of Maci-004’s secondary hydroxyl group under anhydrous conditions produces *tert*-butyldimethylsilyl (TBDMS)-protected derivative Maci-005‌. Controlled oxidation of Maci-005’s primary alcohol functionality using catalytic TEMPO/bleach conditions yields carboxylic acid intermediate Maci-006‌. The synthesis concludes with HATU-mediated amidation of Maci-006 with a sterically hindered amine, achieving neuropeptide mimetic Macimorelin‌.

### Tezacaftor (Symdeko)

Tezacaftor, in combination with ivacaftor as Symdeko, was first approved by the FDA in 2018 for the treatment of cystic fibrosis (CF) in patients aged 12 years and older who are homozygous for the F508del mutation or have at least one mutation in the CFTR gene responsive to Tezacaftor/Ivacaftor based on *in vitro* data or clinical evidence^47^. It is a CFTR “corrector” small molecule, which helps misfolded CFTR protein traffic to the cell surface rather than being degraded, improving the amount of functional CFTR chloride channel at the membrane. Mechanistically, Tezacaftor binds to mutant CFTR protein in the endoplasmic reticulum and helps stabilise its folding, improving its processing, and increasing cell-surface expression, thereby augmenting the function of CFTR, especially when paired with ivacaftor, a potentiator that increases gating of the channel^48^. In preclinical pharmacodynamics, Tezacaftor demonstrated *in vitro* correction of CFTR function in cell lines carrying the F508del mutation, improved chloride transport assays, favourable pharmacokinetics for steady state dosing, and compatibility in combination with ivacaftor regarding safety and exposure. The efficacy of Tezacaftor in combination with ivacaftor was established in the EVOLVE trial, a Phase III, double-blind, placebo-controlled study involving 510 participants with CF homozygous for the F508del mutation. The combination therapy significantly improved lung function and reduced pulmonary exacerbations compared to placebo^48^. Tezacaftor has been evaluated in a Phase 3, open-label study (NCT05844449), which aims to evaluate the long-term safety and efficacy of the Vanzacaftor/Tezacaftor/Deutivacaftor triple combination therapy in cystic fibrosis subjects aged 1 year and older. Common adverse effects include headache, nausea, sinus congestion, and dizziness. The combination therapy has a favourable safety profile, with fewer respiratory-related adverse events compared to previous CFTR modulators^48^.

**Scheme 9.**
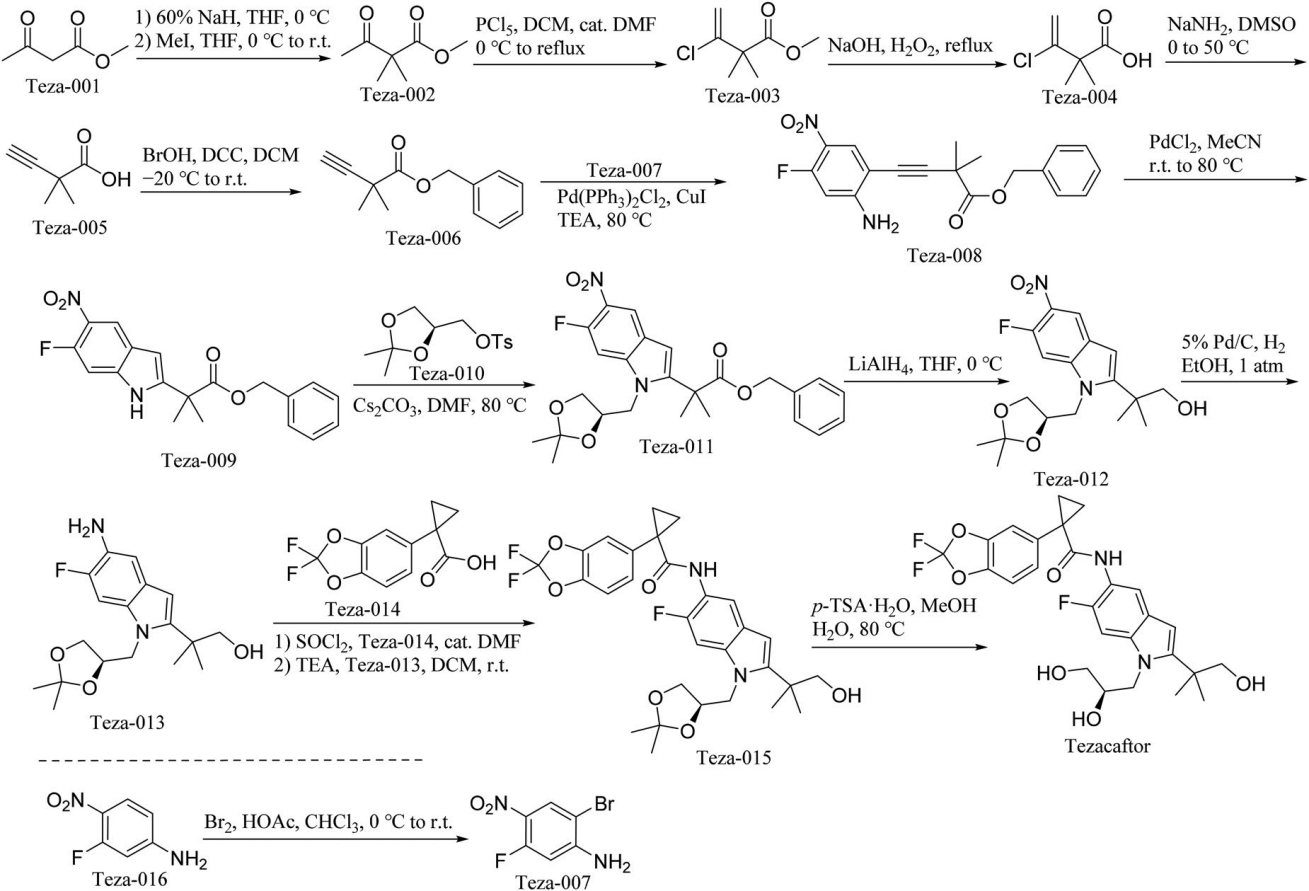
Synthesis of Tezacaftor.

The synthetic route of Tezacaftor is shown in Scheme 9^49^. The synthetic pathway initiates with ‌sodium hydride-activated methylation‌ of Teza-001 to generate methylated intermediate Teza-002‌. Sequential ‌Wittig olefination‌ and ‌chlorination‌ of Teza-002 yield chlorinated compound Teza-003‌. ‌Ester hydrolysis‌ of Teza-003 under aqueous acidic conditions produces carboxylic acid derivative Teza-004‌. Subsequent ‌*β*-elimination‌ of Teza-004 forms *α*,*β*-unsaturated intermediate Teza-005‌. ‌Nucleophilic substitution‌ of Teza-005 introduces functional group diversity, yielding Teza-006‌. Cross-coupling between Teza-006 and brominated partner Teza-007 (synthesised *via* ‌electrophilic bromination‌ of Teza-016‌) generates biaryl-linked Teza-008‌. ‌Thermal [4 + 2] cycloaddition‌ of Teza-008 constructs the fused-ring system in Teza-009‌. A second ‌nucleophilic displacement‌ with Teza-010 installs key heteroatom functionality, forming Teza-011‌. Sequential ‌lithium aluminium hydride-mediated reduction‌ and ‌palladium-catalyzed hydrogenation‌ of Teza-011 achieve stereoselective saturation, producing diastereomerically pure Teza-013‌. ‌Buchwald-Hartwig amination‌ of Teza-013 with Teza-014 introduces the final nitrogen-containing moiety, yielding penultimate intermediate Teza-015‌. ‌Regioselective hydrolysis‌ of Teza-015 under controlled pH conditions completes the synthesis of Tezacaftor‌1.

‌Parallel Synthesis‌: Teza-007 is independently synthesised via ‌radical-mediated bromination‌ of Teza-016 using *N*-bromosuccinimide under photolytic conditions‌.

### Bremelanotide (Vyleesi)

Bremelanotide was approved by the FDA in 2019 for the treatment of acquired, generalised hypoactive sexual desire disorder (HSDD) in premenopausal women^50^. Bremelanotide is a non-selective agonist of melanocortin receptors, primarily targeting melanocortin 3 receptor (MC3R) and melanocortin 4 receptor (MC4R). Activation of these receptors in the central nervous system is believed to modulate pathways associated with sexual desire and arousal, although the exact mechanism remains unclear^51^. Mechanistically, Bremelanotide is a synthetic peptide analog of *α*-melanocyte stimulating hormone (*α*-MSH), thought to activate central melanocortin pathways (notably via MC4R in hypothalamus and other brain areas), modulating sexual desire by enhancing neural signalling of sexual motivation; the precise downstream mechanisms in humans are not fully defined^50^. In preclinical pharmacodynamics, animal studies showed that Bremelanotide increased sexual solicitation and arousal behaviours in rodent models, had good peptide stability, and subcutaneous dosing achieved exposures that activate MC4R pathways; safety pharmacology revealed transient cardiovascular effects in animal models^50^. The efficacy of Bremelanotide was evaluated in two pivotal Phase III clinical trials, identified as RECONNECT studies NCT02333071 and NCT02338960. These randomised, double-blind, placebo-controlled trials demonstrated that Bremelanotide significantly improved sexual desire and reduced distress related to low sexual desire in premenopausal women with HSDD^52^. Bremelanotide has been evaluated in a Phase 4 study (NCT04179734). This trial aimed to investigate the involvement of the melanocortin-4 receptor in HSDD and assess the efficacy of Bremelanotide in treating this condition. Common adverse effects of Bremelanotide include nausea, flushing, headache, and injection site reactions. Notably, nausea was most prevalent after the first dose and typically lasted about 2 h. Other side effects may include transient increases in blood pressure and decreases in heart rate.

**Scheme 10.**
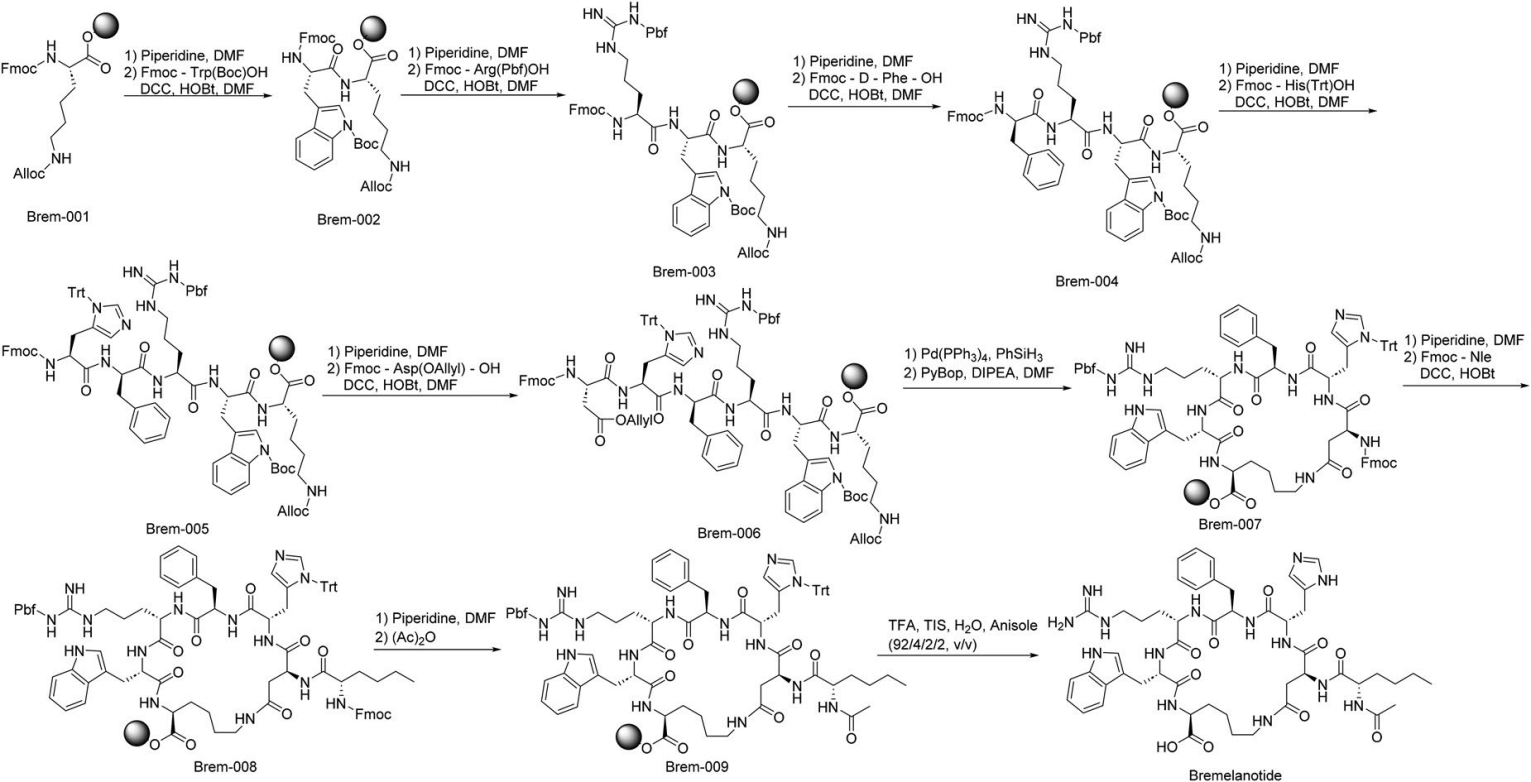
Synthesis of Bremelanotide.

The synthetic route of Bremelanotide is shown in Scheme 10^53^. The synthesis commences with ‌Fmoc deprotection‌ of Brem-001 under mild basic conditions‌. Sequential ‌Fmoc-XX group condensations‌ *via* carbodiimide coupling reagents progressively assemble the peptide backbone, yielding intermediate Brem-005‌.‌ Palladium-mediated allyl cleavage‌ of Brem-005 generates free thiol-containing Brem-006‌. Subsequent ‌thioester-directed condensation‌ with activated carboxylates forms disulfide-bridged Brem-007‌. Piperidine-driven Fmoc removal‌ followed by ‌acetylation‌ with acetic anhydride produces N-acetylated Brem-008. HATU-mediated amidation‌ with protected amines yields cyclized intermediate Brem-009‌. Final ‌global deprotection‌ of Brem-009 using optimised cleavage cocktails releases Bremelanotide‌.

### Flortaucipir F18 (Tauvid)

Flortaucipir F18, marketed under the brand name Tauvid, is a radioactive diagnostic agent developed by Avid Radiopharmaceuticals, a subsidiary of Eli Lilly and Company^54^. The FDA approved Tauvid in 2020 for positron emission tomography (PET) imaging to estimate the density and distribution of aggregated tau neurofibrillary tangles (NFTs) in adult patients with cognitive impairment who are being evaluated for Alzheimer’s disease (AD). Flortaucipir F18 binds selectively to aggregated tau protein, specifically paired helical filament (PHF) tau, with a dissociation constant (*K_d_*) of 0.57 nM. In the brains of patients with AD, tau aggregates combine to form NFTs, one of the two components required for the neuropathological diagnosis of AD. *In vitro* studies demonstrated that Flortaucipir F18 binds to PHF tau purified from brain homogenates of donors with AD, indicating its potential for detecting tau pathology *in vivo*. The efficacy of Flortaucipir F18 was evaluated in a pivotal Phase III trial (NCT03901092). This study assessed the correlation between Flortaucipir PET imaging and post-mortem assessments of tau pathology, demonstrating that Flortaucipir PET scans could accurately identify tau pathology in patients being evaluated for AD^55^. Flortaucipir F18 has been evaluated in a Phase III study (NCT04437511). This double-blind, placebo-controlled trial assessed the safety and efficacy of donanemab in participants with early symptomatic Alzheimer’s disease. A secondary outcome measure of this study was the change from baseline in brain tau deposition as measured by Flortaucipir F18 PET scans. Administration of Flortaucipir F18 was generally safe, with relatively few adverse effects reported among patients enrolled in clinical studies. Some patients experienced minor side effects such as injection site reactions and mild systemic symptoms. Rarely, more severe allergic reactions can occur, and there is a general risk associated with radiation exposure^56^.

**Scheme 11.**
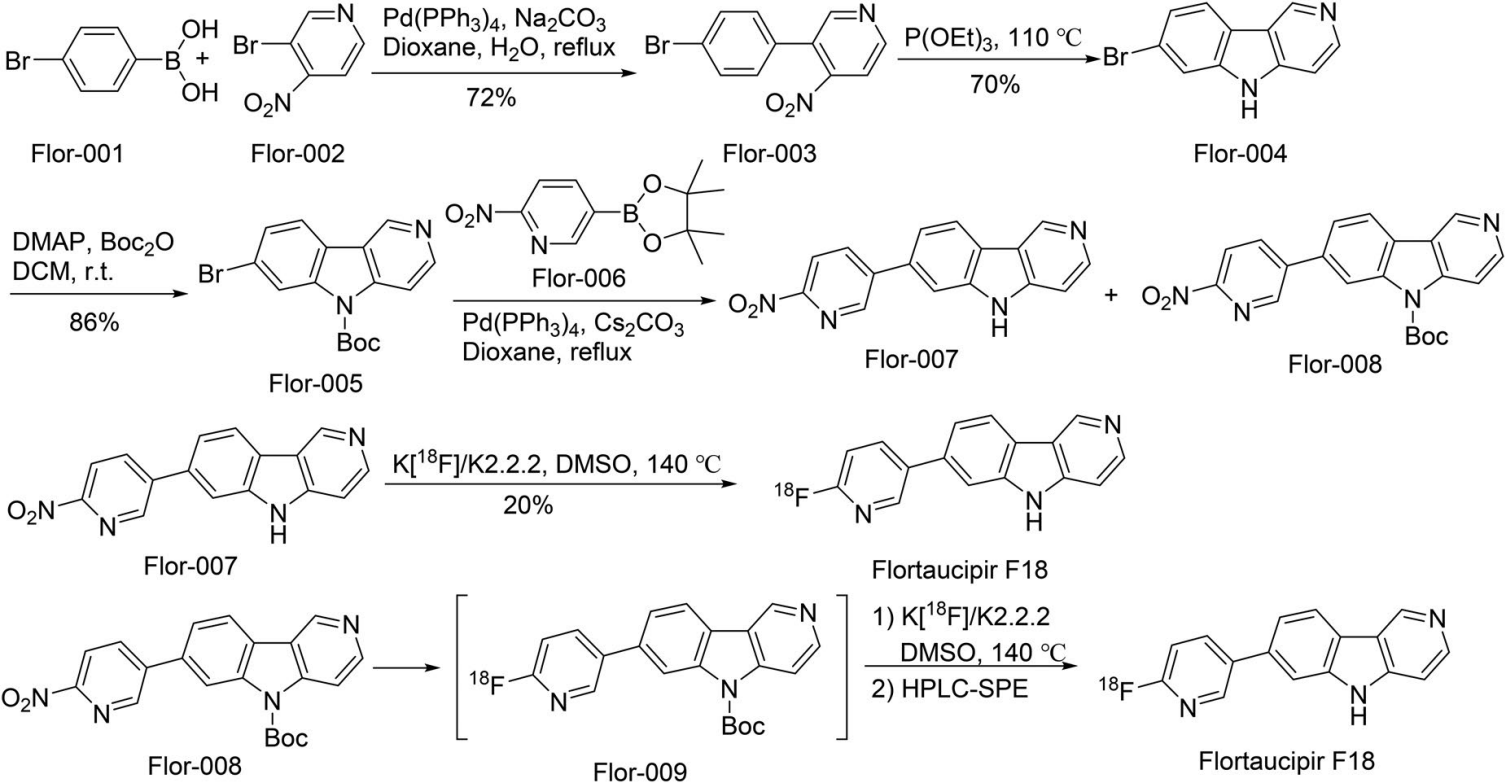
Synthesis of Flortaucipir F18.

The synthetic route of Flortaucipir F18 is shown in Scheme 11^57^. The synthetic route initiates with a ‌palladium-catalyzed Suzuki-Miyaura cross-coupling‌ between Flor-001 and Flor-002 to afford biaryl intermediate Flor-003‌. Subsequent ‌catalytic hydrogenative cyclization‌ of Flor-003 generates the fused-ring scaffold Flor-004‌. A ‌strategic hydroxyl protection‌ step using *tert*-butyldimethylsilyl (TBS) chloride converts Flor-004 into silyl-protected Flor-005. Flor-005 undergoes a ‌regioselective Suzuki-Miyaura coupling‌ with boronic ester Flor-006, yielding regioisomeric intermediates Flor-007 and Flor-008. Fluorine-18 nucleophilic displacement‌ on Flor-007 under anhydrous conditions directly produces radiolabeled Flortaucipir F18. Chloride-to-amine substitution‌ of Flor-008 with aqueous ammonia generates advanced intermediate Flor-009‌. Final ‌acid-mediated TBS deprotection‌ of Flor-009 completes the synthesis of Flortaucipir F18, achieving both radiochemical purity and structural fidelity‌.

### Lurbinectedin (Zepzelca)

Lurbinectedin, marketed as Zepzelca, is a synthetic derivative of trabectedin, which is indicated for the treatment of adult patients with metastatic small cell lung cancer (SCLC) who have experienced disease progression on or after platinum-based chemotherapy^58^. The FDA granted accelerated approval in 2020. Lurbinectedin binds to the minor groove of DNA, causing DNA strand breaks and inhibiting RNA polymerase II activity. This disruption leads to the downregulation of oncogenic transcription factors, resulting in cell cycle arrest and apoptosis in tumour cells. In preclinical studies, Lurbinectedin demonstrated potent antitumor activity against various cancer cell lines, particularly those with defects in DNA repair mechanisms. Its ability to induce DNA damage and apoptosis was evident in models of SCLC^59^. Clinical efficacy in the pivotal single-arm B-005-14 (PM1183-B-005-14) study (NCT02454972) in 105 patients showed an overall response rate (ORR) of ∼35% (with median duration of response ∼5.3 months), leading to its approval. Regarding toxicity, common adverse events include myelosuppression, fatigue, nausea, decreased appetite, elevated hepatic transaminases; serious adverse events included febrile neutropenia, dose reductions and delays, discontinuations, but no treatment-related deaths in the trial.

**Scheme 12.**
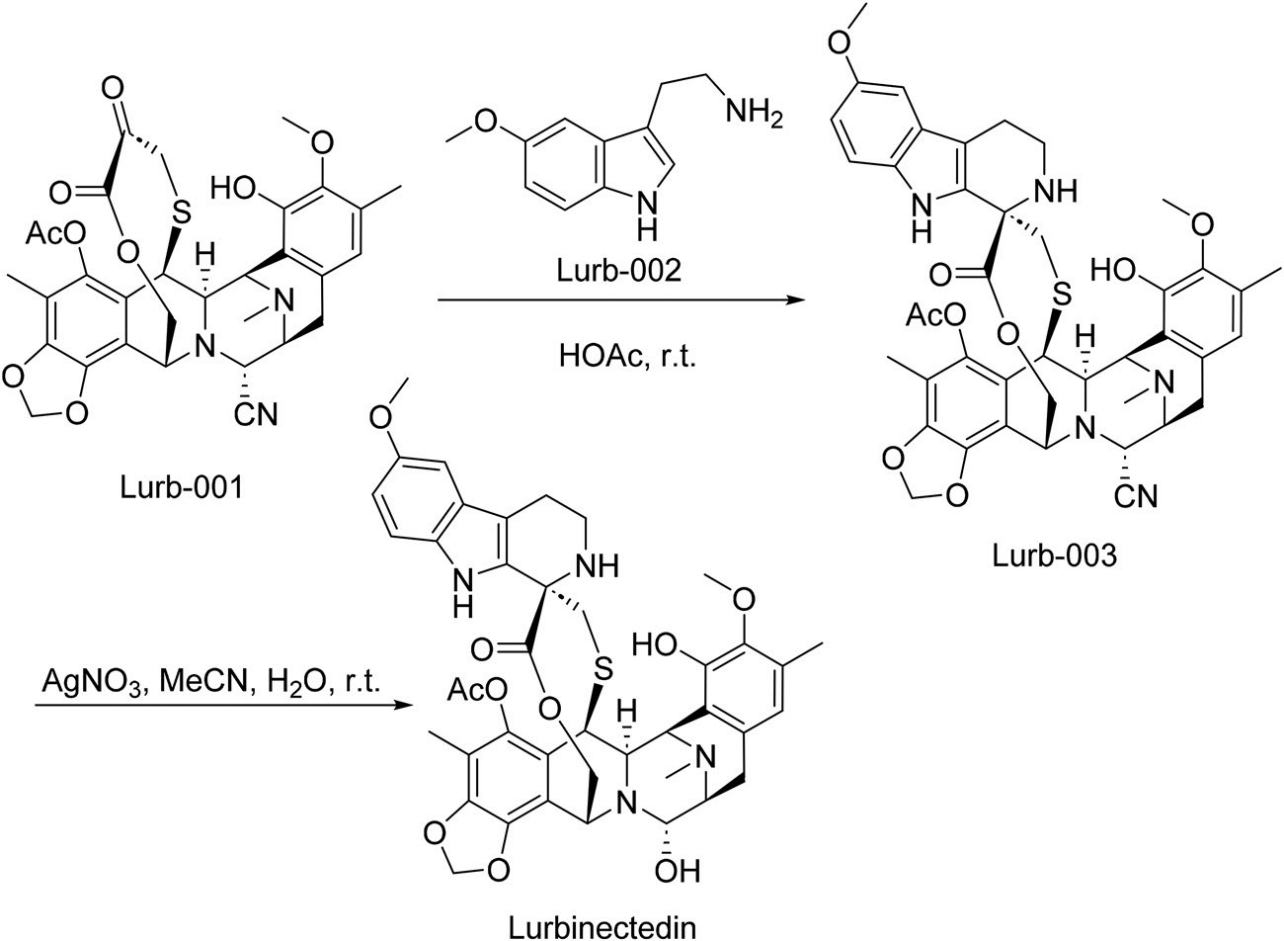
Synthesis of Lurbinectedin.

The synthetic route of Lurbinectedin is shown in Scheme 12^60^. Lurb-001 and Lurb-002 participate in a nucleophilic substitution reaction, through which Lurb-003 is synthesised. Subsequently, Lurb-003 undergoes an oxidation reaction, successfully culminating in the synthesis of Lurbinectedin.

### Setmelanotide (Imcivree)

Setmelanotide, marketed under the brand name Imcivree, is a melanocortin-4 receptor (MC4R) agonist^61^. The FDA first approved Imcivree in 2020 for chronic weight management in adult and paediatric patients aged 6 years and older with obesity due to pro-opiomelanocortin (POMC), proprotein convertase subtilisin/kexin type 1 (PCSK1), or leptin receptor (LEPR) deficiencies. Subsequently, in 2022, the FDA expanded the approval to include patients with Bardet-Biedl syndrome (BBS). In 2024, the FDA further extended the indication to include patients as young as 2 years old with these conditions^62^. Setmelanotide functions as an MC4R agonist, activating the receptor in place of alpha-melanocyte-stimulating hormone (*α*-MSH). This activation re-establishes MC4R pathway activity, which is crucial for regulating hunger, satiety, and energy expenditure. By restoring this pathway, Setmelanotide helps reduce hunger and promote weight loss in individuals with specific genetic deficiencies affecting the MC4R pathway. In preclinical studies, Setmelanotide demonstrated the ability to decrease body weight in obese animal models. These effects were associated with significant reductions in adiposity and improvements in glucose tolerance and insulin sensitivity. Importantly, Setmelanotide targets MC4R receptors in the brain without notable effects on central nervous system parameters, including motor activity or respiratory function. The efficacy of Setmelanotide was evaluated in the EMANATE trial (NCT05093634), a Phase III study involving patients with specific gene variants in the MC4R pathway. The trial demonstrated that Setmelanotide led to significant weight loss and reductions in hunger scores among participants. Setmelanotide has been evaluated in a Phase III study (NCT05194124). This trial assessed the safety and efficacy of a weekly depot formulation of Setmelanotide in patients with obesity due to specific genetic deficiencies. The study aimed to provide a more convenient dosing regimen compared to the daily administration of Setmelanotide. Common adverse effects of Setmelanotide include injection site reactions, skin hyperpigmentation, nausea, headache, and diarrhoea. These side effects are generally transient and manageable.

**Scheme 13.**
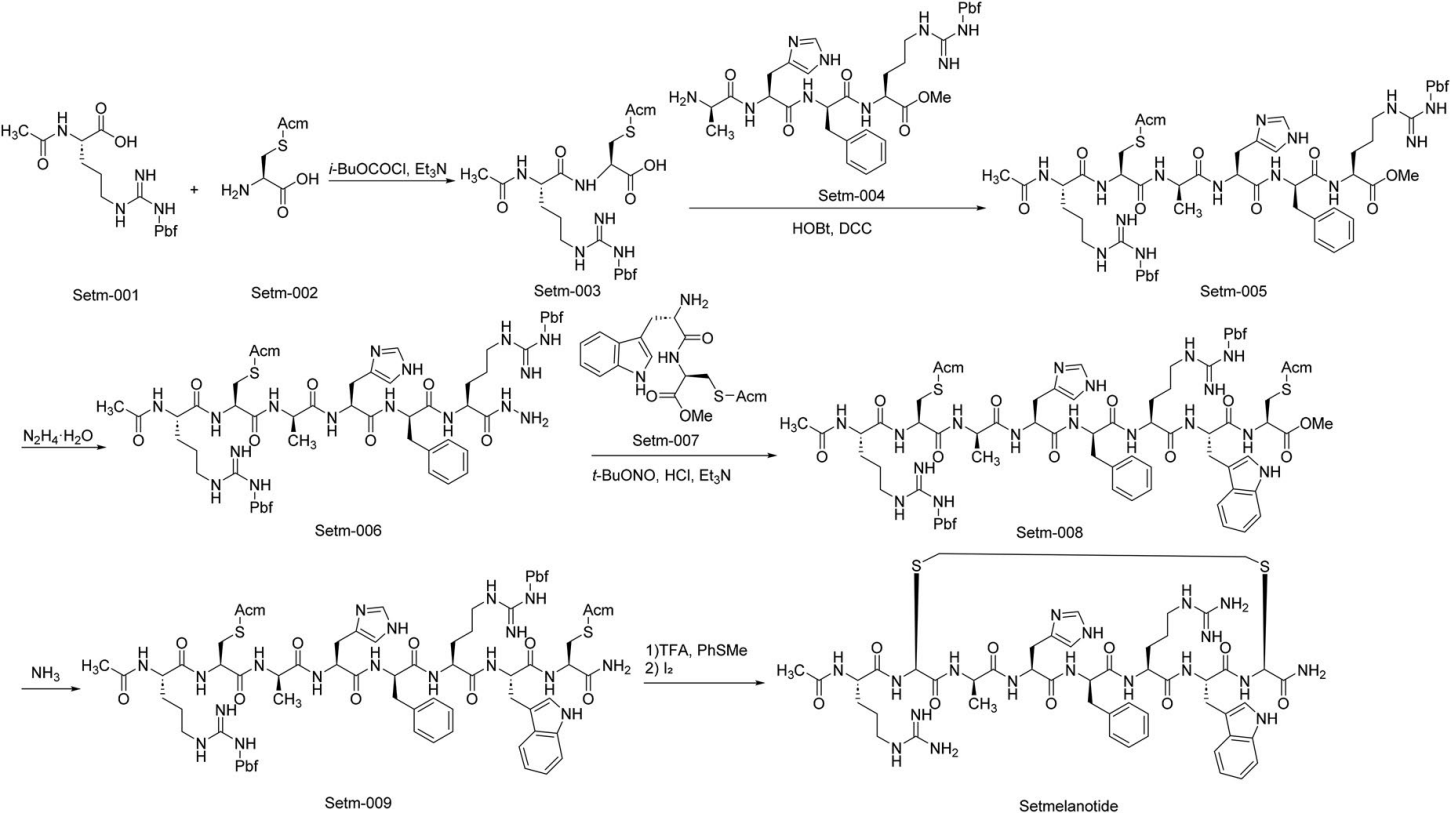
Synthesis of Setmelanotide.

The synthetic route of Setmelanotide is shown in Scheme 13^63^. The synthetic sequence initiates with a ‌carbodiimide-mediated condensation‌ between Setm-001 and Setm-002 to yield peptide intermediate Setm-003‌. Subsequent ‌amide coupling‌ of Setm-003 with carboxylic acid derivative Setm-004 produces extended-chain intermediate Setm-005‌. A ‌sequential amidation strategy‌ is employed, where Setm-005 undergoes further coupling with activated amino acid derivatives to generate Setm-006‌. Controlled ‌oxidative functionalization‌ of Setm-006 with Setm-007 under inert atmosphere yields disulfide-linked dimer Setm-008‌. Setm-008 then participates in a ‌regioselective amidation‌ to install critical side-chain functionality, forming advanced intermediate Setm-009‌. The synthesis culminates in a ‌one-pot deprotection-cyclisation cascade‌, where acid-labile protecting groups are selectively cleaved while simultaneously inducing macrocyclic ring formation through intramolecular nucleophilic attack, ultimately affording Setmelanotide‌.

### Mobocertinib (Exkivity)

Mobocertinib, marketed as Exkivity, is an oral TKI^64^. The FDA granted accelerated approval in 2021 for the treatment of adult patients with locally advanced or metastatic NSCLC harbouring EGFR exon 20 insertion mutations, whose disease has progressed on or after platinum-based chemotherapy^65^. Mobocertinib is designed to selectively target and irreversibly bind to mutant EGFR proteins with exon 20 insertions, inhibiting their kinase activity and thereby blocking downstream signalling pathways that promote tumour cell proliferation. In preclinical studies, Mobocertinib demonstrated potent inhibition of EGFR exon 20 insertion mutations, leading to significant antitumor activity in cell-based assays and animal models^66^. The efficacy of Mobocertinib was evaluated in a Phase I/II trial (NCT02716116), involving patients with EGFR exon 20 insertion mutation-positive metastatic NSCLC who had received prior platinum-based chemotherapy. The study reported an objective response rate (ORR) of 28%, with a median duration of response of 17.5 months. Common adverse reactions include diarrhoea, rash, nausea, stomatitis, vomiting, decreased appetite, paronychia, fatigue, dry skin, and musculoskeletal pain. Serious adverse effects may involve QTc prolongation, cardiac toxicity, and interstitial lung disease.

**Scheme 14.**
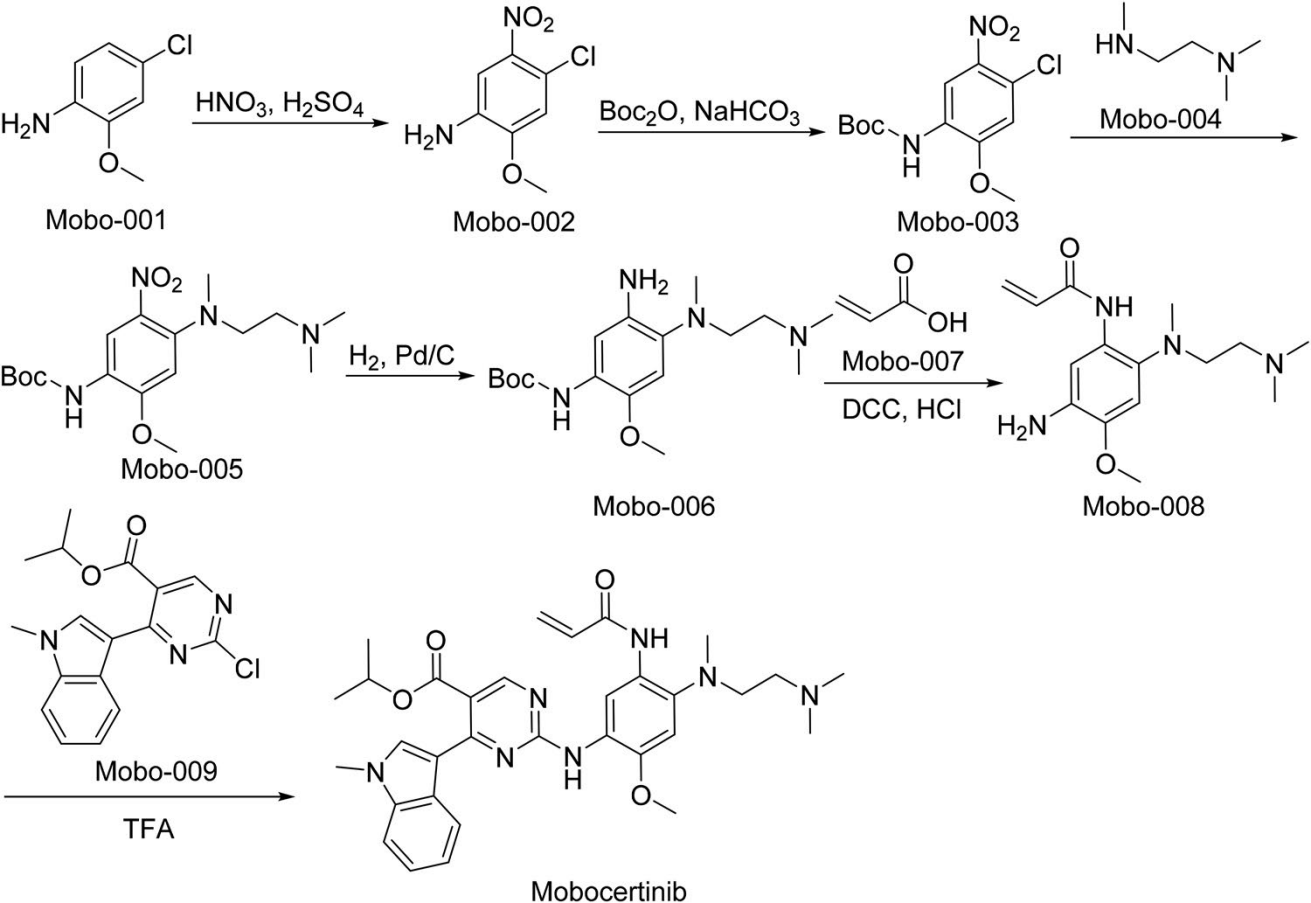
Synthesis of Mobocertinib.

The synthetic route of Mobocertinib is shown in Scheme 14^67^. The synthetic sequence initiates with ‌aromatic nitration‌ of Mobo-001 to yield nitro-substituted intermediate Mobo-002‌. Subsequent ‌nucleophilic aromatic substitution‌ introduces a halogen leaving group, forming aryl amine derivative Mobo-003‌. ‌Alkylative substitution‌ with nucleophile Mobo-004 installs a branched alkyl chain, generating Mobo-005‌.‌ Catalytic nitro group reduction‌ converts Mobo-005 to the corresponding amine intermediate Mobo-006‌. A ‌concomitant nucleophilic substitution-amidation‌ with carboxylic acid derivative Mobo-007 establishes the amide linkage in Mobo-008‌. The synthesis concludes through ‌stereospecific SN_2_ displacement‌ with chiral electrophile Mobo-009, achieving Mobocertinib‌.

### Etrasimod (Velsipity)

Etrasimod (Velsipity) was FDA-approved in 2023 for treating moderately to severely active ulcerative colitis in adults based on the ELEVATE UC 12 and 52 Phase III trials^68^. It is a selective small-molecule modulator of sphingosine-1-phosphate receptors S1P_1_, S1P_4_, and S1P_5_ (minimal activity on S1P_3_, none on S1P_2_) acting by reducing egress of lymphocytes from lymphoid organs to lower peripheral blood lymphocyte counts and thereby diminishing lymphocyte infiltration in the gut mucosa. In preclinical pharmacodynamics it shows full agonist activity at human S1P_1_ (EC_50_ ∼6.1 nM), mouse (∼3.65 nM), dog (∼4.19 nM), monkey (∼8.7 nM), partial agonism at human S1P_4_ (≈147 nM, ∼63% efficacy) and S1P_5_ (≈24.4 nM, ∼73% efficacy), no activity at S1P_2_/S1P_3_, dose-dependent reduction in lymphocyte count in mice, and efficacy in experimental colitis models^69^. Clinical efficacy was demonstrated in ELEVATE UC 52 and 12 where once-daily 2 mg Etrasimod produced remission rates *versus* placebo (∼27 *vs.* ∼7% at week 12; ∼32 *vs.* ∼7% at week 52 in UC-52; ∼26 *vs.* ∼15% at week 12 in UC-12), with statistically significant improvements in secondary endpoints like endoscopic healing, sustained remission, mucosal healing, corticosteroid-free remission; toxicity was generally mild-to-moderate, common adverse events included headache, elevated liver tests, dizziness, abdominal pain, nausea, worsening UC, COVID-19 infection, arthralgia; transient decreases in heart rate and AV conduction when initiating treatment; risk of QT prolongation especially with concomitant QT-prolonging drugs; animal preclinical studies showed foetal harm at high multiples of human exposure, some hemangiosarcoma/hemangioma in long-term carcinogenicity studies in mice at very high doses, and reproductive toxicity at exposures many times the MRHD.

**Scheme 15.**
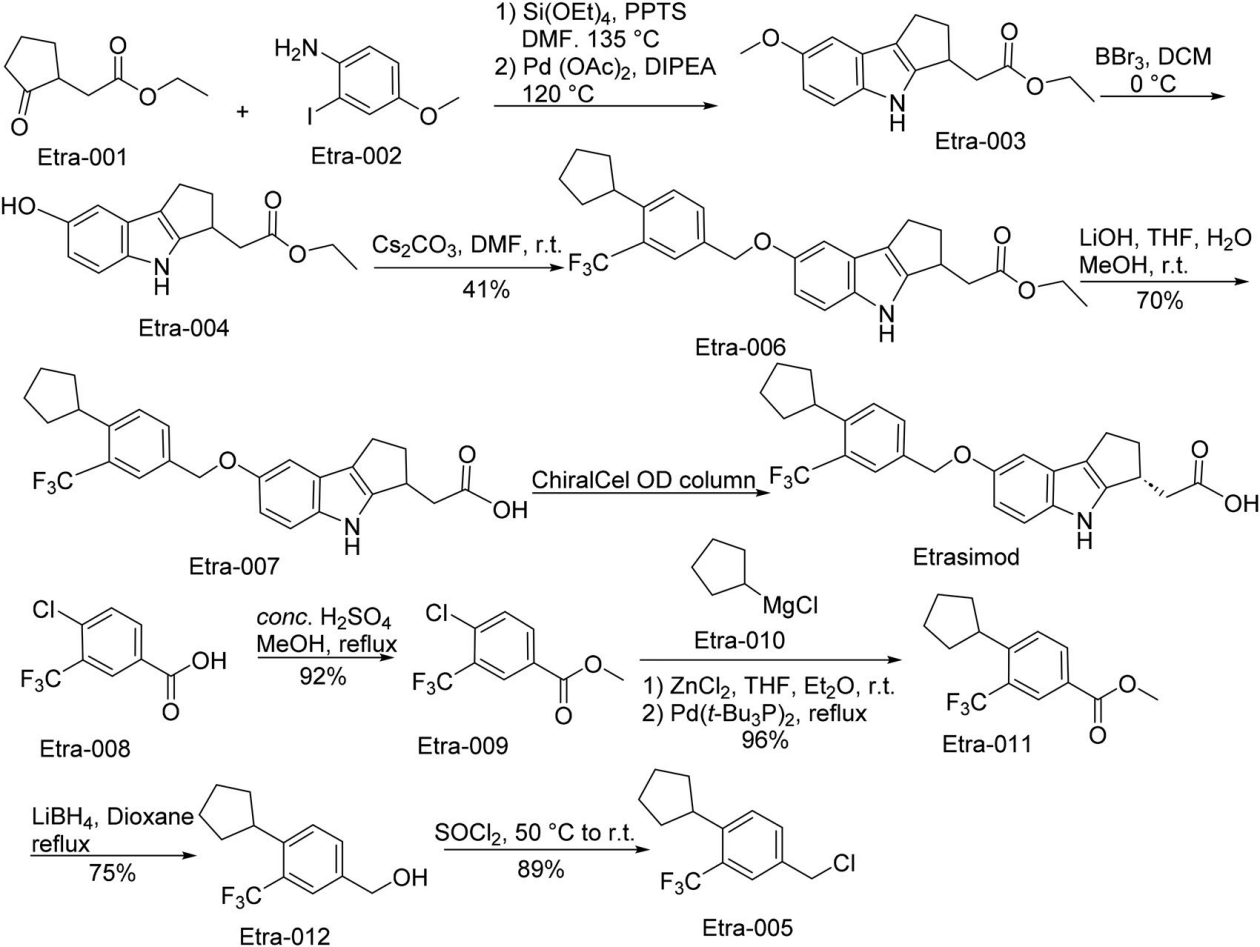
Synthesis of Etrasimod.

The synthetic route of Etrasimod is shown in Scheme 15^70–72^. The synthesis initiates with a ‌Michael addition-condensation cascade‌ between Etra-001 and Etra-002, yielding bicyclic intermediate Etra-003‌. Subsequent ‌acid-catalyzed demethylation‌ of Etra-003 generates phenolic derivative Etra-004‌. Etra-004 undergoes ‌SN_2_ displacement‌ with Etra-005 under mild basic conditions to form ether-linked Etra-006‌. Controlled ‌alkaline hydrolysis‌ of Etra-006 in aqueous lithium hydroxide selectively cleaves the ester moiety, producing carboxylic acid intermediate Etra-007‌. ‌Chiral resolution‌ of Etra-007 *via* preparative chromatography isolates the target enantiomer, Etrasimod arginine‌.‌ In parallel, Etra-008 undergoes ‌acid-mediated esterification‌ with methanol to yield methyl ester Etra-009‌. A ‌Grignard reagent-mediated alkylation‌ of Etra-009 with Etra-010 generates tertiary alcohol Etra-011‌. ‌Catalytic hydrogenation‌ of Etra-011 reduces carbonyl groups to methylene units, producing Etra-012‌. Finally, ‌thionyl chloride-assisted chlorination‌ converts Etra-012 into reactive chloride Etra-005, closing the synthetic loop‌.

### Iptacopan (Fabhalta)

Iptacopan, marketed under the brand name Fabhalta, is an oral complement factor B inhibitor^73^. It received its first approval from the FDA in 2023 for the treatment of adults with paroxysmal nocturnal haemoglobinuria (PNH). Iptacopan binds to factor B of the alternative complement pathway, inhibiting its activity. This action prevents the cleavage of complement component C3, thereby reducing the formation of downstream effectors and the amplification of the terminal complement pathway. In PNH, this inhibition controls both C3b-mediated extravascular haemolysis and terminal complement-mediated intravascular haemolysis. In preclinical studies, Iptacopan demonstrated potent and selective inhibition of the alternative complement pathway by targeting factor B. This inhibition effectively reduced complement activation, suggesting potential therapeutic benefits in complement-mediated diseases. The efficacy of Iptacopan was evaluated in the APPLY-PNH (NCT04558918). A randomised, open-label study comparing Iptacopan to standard-of-care anti-C5 therapies in adults with PNH. Results indicated that Iptacopan provided superior haemoglobin improvement without the need for transfusions. Common adverse reactions to Iptacopan in PNH patients include headache, nasopharyngitis, diarrhoea, abdominal pain, bacterial infections, viral infections, nausea, and rash.

**Scheme 16.**
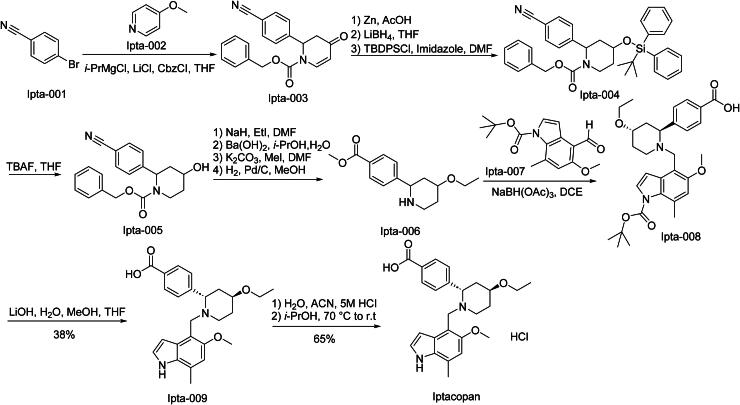
Synthesis of Iptacopan.

The synthetic route of Iptacopan is shown in Scheme 16^74^. Ipta-001 and Ipta-002 participate in nucleophilic substitution and Grignard reaction for coupling, leading to the synthesis of Ipta-003. Subsequently, Ipta-003 undergoes reduction and silanization reactions to yield Ipta-004. Ipta-004 then experiences hydroxylation to remove the protecting group, thereby synthesising Ipta-005. Ipta-005 goes through a series of reactions, namely alkylation, transesterification, and reduction, to form Ipta-006. After that, Ipta-006 and Ipta-007 undergo a condensation reaction to produce Ipta-008. Ipta-008 further undergoes a deprotection reaction to generate Ipta-009. Finally, Ipta-009 is acidified to synthesise Iptacopan.

## Discussion and conclusion

The collective analysis of FDA-approved indole-containing drugs highlights not only the privileged nature of the indole scaffold but also the evolving toolbox required to exploit it effectively in modern drug discovery. Numerous recent reviews reiterate that indole remains one of the most frequently used heterocycles in approved drugs, owing to its ability to engage diverse biological targets through *π*–*π* stacking, hydrogen bonding, and cation–*π* interactions, and to mimic key endogenous motifs such as tryptophan and serotonin^3^. However, “privileged” does not mean “problem-free”: indole derivatives routinely force medicinal chemists to confront issues of metabolic soft spots, off-target binding, and suboptimal developability.

From a synthetic perspective, there has been a clear shift from classical, linear routes towards more agile strategies that support rapid structure–activity relationship exploration and late-stage diversification. Recent advances in late-stage functionalization (LSF) and C–H activation have been particularly impactful, enabling direct modification of complex indole cores without the need for *de novo* synthesis. Comprehensive surveys of LSF and C–H functionalization in drugs and drug-like molecules show that these methods can dramatically accelerate lead optimisation, allowing medicinal chemists to modulate polarity, lipophilicity, and target engagement by installing small fragments at otherwise inaccessible positions^75^. In parallel, work on late-stage meta-C(sp^2^)–H alkylation and directed arene functionalization in pharmaceutical scaffolds illustrates how strategically placed directing groups can unlock regioselective modifications on heteroaromatics, including indoles, in a single step^76^. These technologies are directly aligned with the needs identified in our case studies, where fine-tuning of indole substitution patterns often translated into improved potency, selectivity, or pharmacokinetic properties.

However, metabolic liability remains a recurring challenge. The electron-rich indole ring is particularly prone to oxidative metabolism (e.g. aromatic hydroxylation and N-oxidation), which can accelerate clearance or generate reactive metabolites. Broader analyses of heterocycle metabolism and biotransformation emphasise that such vulnerabilities are a major cause of late-stage attrition and motivate early integration of metabolite-profiling and “soft-spot” identification in lead optimisation^77^. Strategies to mitigate these liabilities include scaffold hopping, judicious incorporation of electron-withdrawing substituents, or bioisosteric replacement of particularly labile indole positions, as exemplified by recent work using scaffold changes to address oxidative metabolism in aromatic systems^78^. Coupling such structural tactics with *in silico* metabolism and toxicity prediction, now increasingly supported by machine-learning models, can help prioritise indole analogs with more favourable ADME/T profiles before they reach advanced development^79^.

The importance of balancing target potency with safety and tolerability should also be noticed. Reviews of indole-based therapeutics show that the same physicochemical traits that favour strong binding (planarity, aromaticity, lipophilicity) can also drive promiscuity and off-target effects if not carefully managed^80^. By temporarily masking polar functionalities or modulating release profiles, prodrugs and formulation strategies can improve bioavailability, reduce peak systemic exposure, and localise pharmacological action, particularly relevant for CNS-active or oncology-focused indole drugs where therapeutic indices are narrow.

Looking forward, several opportunities emerge: The growing palette of metal-catalyzed, metal-free, and photoredox-based LSF methods is ideally suited for generating second-generation analogs and exploring life-cycle management around already approved indole drugs^81^. For anti-infective and anticancer indole agents, where resistance is a concern, integration of structural biology and resistance-mapping can guide substitutions on the indole core that maintain activity while bypassing known resistance mechanisms. Recent work on indole-based hybrids (e.g. indole–imidazole frameworks) illustrates how combining indole with a second pharmacophore can deliver multi-target engagement or broaden therapeutic scope, an approach that could be extended to FDA-approved backbones^82^. Reviews on indole scaffolds consistently emphasise that their success stems from precisely this interplay, where synthetic creativity informs biological hypothesis-testing and clinical needs feedback to refine molecular design^83^. Moreover, existing approved indole drugs represent fertile starting points for life-cycle management, including analog development or repurposing in new indications. Ultimately, by viewing indole-based drug development through an integrated synthetic-to-clinical lens, this review aspires to offer practical insights and guidance to medicinal chemists, pharmacologists, and translational scientists. We anticipate that with continued methodological advances and tighter cross-disciplinary integration, the indole scaffold will remain a powerful and productive platform for next-generation therapeutic discovery.

## Data Availability

Data will be made available on request.

## References

[1] Mo X, Rao DP, Kaur K, Hassan R, Abdel-Samea AS, Farhan SM, Bräse S, Hashem H. Indole derivatives: a versatile scaffold in modern drug discovery-an updated review on their multifaceted therapeutic applications (2020–2024). Molecules. 2024;29(19):4770. 39407697 10.3390/molecules29194770 PMC11477627

[2] Duan SF, Song L, Guo HY, Deng H, Huang X, Shen QK, Quan ZS, Yin XM. Research status of indole-modified natural products. RSC Med Chem. 2023;14(12):2535–2563. 38107170 10.1039/d3md00560g PMC10718587

[3] Kumari A, Singh RK. Medicinal chemistry of indole derivatives: current to future therapeutic prospectives. Bioorg Chem. 2019;89:103021. 31176854 10.1016/j.bioorg.2019.103021

[4] Zeng W, Han C, Mohammed S, Li S, Song Y, Sun F, Du Y. Indole-containing pharmaceuticals: targets, pharmacological activities, and SAR studies. RSC Med Chem. 2024;15(3):788–808. 38516587 10.1039/d3md00677h PMC10953485

[5] Biskobing DM. Update on bazedoxifene: a novel selective estrogen receptor modulator. Clin Interv Aging. 2007;2(3):299–303. 18044180 PMC2685267

[6] Gennari L, Merlotti D, De Paola V, Martini G, Nuti R. Bazedoxifene for the prevention of postmenopausal osteoporosis. Ther Clin Risk Manag. 2008;4(6):1229–1242. 19337430 10.2147/tcrm.s3476 PMC2643104

[7] Miller CP, Tran BD, Collini MD. Preparation of 2-phenyl-1-[4-(2-aminoethoxy)benzyl]indoles as estrogenic agents. American Home Products Corporation; 1997. US5998402A.

[8] Miller CP, Collini MD, Tran BD, Harris HA, Kharode YP, Marzolf JT, Moran RA, Henderson RA, Bender RH, Unwalla RJ, et al. Design, synthesis, and preclinical characterization of novel, highly selective indole estrogens. J Med Chem. 2001;44(11):1654–1657. 11356100 10.1021/jm010086m

[9] Wind S, Schmid U, Freiwald M, Marzin K, Lotz R, Ebner T, Stopfer P, Dallinger C. Clinical pharmacokinetics and pharmacodynamics of nintedanib. Clin Pharmacokinet. 2019;58(9):1131–1147. 31016670 10.1007/s40262-019-00766-0 PMC6719436

[10] Tepede A, Yogaratnam D. Nintedanib for idiopathic pulmonary fibrosis. J Pharm Pract. 2019;32(2):199–206. 29017422 10.1177/0897190017735242

[11] Khalique S, Banerjee S. Nintedanib in ovarian cancer. Expert Opin Investig Drugs. 2017;26(9):1073–1081. 10.1080/13543784.2017.1353599 28721753

[12] Gole S, Bankole A. Nintedanib, StatPearls, StatPearls Publishing Copyright © 2025. Disclosure: Adegbenga Bankole declares no relevant financial relationships with ineligible companies. Treasure Island (FL): StatPearls Publishing LLC; 2025.

[13] Flaherty KR, Wells AU, Cottin V, Devaraj A, Walsh SLF, Inoue Y, Richeldi L, Kolb M, Tetzlaff K, Stowasser S, et al. Nintedanib in progressive fibrosing interstitial lung diseases. N Engl J Med. 2019;381(18):1718–1727. 31566307 10.1056/NEJMoa1908681

[14] Heckel A, Roth GJ, Walter R, Van Meel J, Redemann N, Tontsch-Grunt U, Spevak W, Hilberg F. Preparation of substituted aminomethyleneindolinone inhibitors of tyrosine receptor kinases and CDK/cyclin kinases as antitumor agents and inhibitors of cell proliferation. Boehringer Ingelheim Pharma K.-G.; 2001. WO0127081A1.

[15] Garnock-Jones KP. Panobinostat: first global approval. Drugs. 2015;75(6):695–704. 25837990 10.1007/s40265-015-0388-8

[16] Raedler LA. Farydak (panobinostat): first HDAC inhibitor approved for patients with relapsed multiple myeloma. Am Health Drug Benefits. 2016;9:84–87. PMC5013857 27668050

[17] Hennika T, Hu G, Olaciregui NG, Barton KL, Ehteda A, Chitranjan A, Chang C, Gifford AJ, Tsoli M, Ziegler DS, et al. Pre-clinical study of panobinostat in xenograft and genetically engineered murine diffuse intrinsic pontine glioma models. PLOS One. 2017;12(1):e0169485. 28052119 10.1371/journal.pone.0169485 PMC5215670

[18] Richardson PG, Hungria VTM, Yoon S-S, Beksac M, Dimopoulos MA, Elghandour A, Jedrzejczak WW, Guenther A, Na Nakorn T, Siritanaratkul N, et al. Panorama 1: a randomized, double-blind, phase 3 study of panobinostat or placebo plus bortezomib and dexamethasone in relapsed or relapsed and refractory multiple myeloma. J Clin Oncol. 2014;32(15_suppl):8510.

[19] LiverTox: clinical and research information on drug-induced liver injury. Bethesda (MD): National Institute of Diabetes and Digestive and Kidney Diseases; 2012. 31643176

[20] Santos LS, Pilli RA, Rawal VH. Enantioselective total syntheses of (+)-arborescidine A, (−)-arborescidine B, and (−)-arborescidine C. J Org Chem. 2004;69(4):1283–1289. 14961682 10.1021/jo035165f

[21] Izumo S, Shetty SS. Preparation of cinnamic acid N-hydroxy amides as histone deacetylase inhibitors for the treatment of pathologic cardiac hypertrophy and heart failure. Novartis A.-G. Novartis Pharma G.m.b.H.; 2007. WO2007021682A1.

[22] Malapelle U, Ricciuti B, Baglivo S, Pepe F, Pisapia P, Anastasi P, Tazza M, Sidoni A, Liberati AM, Bellezza G, et al. Osimertinib, recent results in cancer research. Fortschritte der Krebsforschung. Recent Results Cancer Res. 2018;211:257–276. 30069773 10.1007/978-3-319-91442-8_18

[23] Greig SL. Osimertinib: first global approval. Drugs. 2016;76(2):263–273. 26729184 10.1007/s40265-015-0533-4

[24] Zhang Y, Liu X, Zhang C, Li Y, Zhang G, Du Z. FDA-approved pyrimidine-containing drugs: synthesis and clinical application. Med Res Rev. 2025. 10.1002/med.70027. 41267351

[25] Remon J, Steuer CE, Ramalingam SS, Felip E. Osimertinib and other third-generation EGFR TKI in EGFR-mutant NSCLC patients. Ann Oncol. 2018;29(suppl_1):i20–i27. 29462255 10.1093/annonc/mdx704

[26] Odogwu L, Mathieu L, Goldberg KB, Blumenthal GM, Larkins E, Fiero MH, Rodriguez L, Bijwaard K, Lee EY, Philip R, et al. FDA benefit-risk assessment of osimertinib for the treatment of metastatic non-small cell lung cancer harboring epidermal growth factor receptor T790M mutation. Oncologist. 2018;23(3):353–359. 29242281 10.1634/theoncologist.2017-0425 PMC5905690

[27] Ji M, Li Y, Liu H, Li R, Cai J, Hu H. Synthetic method of N-[2-[[2-(dimethylamino)ethyl]methylamino]-4-methoxy-5-[[4-(1-methyl-1H-indol-3-yl)-2-pyrimidinyl]amino]phenyl]-2-acrylamide as antitumor drug. Suzhou Southeast Pharmaceuticals Co., Ltd.; 2015. CN104817541A.

[28] Herden M, Waller CF. Alectinib. Recent Results Cancer Res. 2018;211:247–256. 30069772 10.1007/978-3-319-91442-8_17

[29] Peters S, Camidge DR, Shaw Alice T, Gadgeel S, Ahn Jin S, Kim D-W, Ou Sai-Hong I, Pérol M, Dziadziuszko R, Rosell R, et al. Alectinib versus crizotinib in untreated ALK-positive non-small-cell lung cancer. N Engl J Med. 2017;377(9):829–838. 28586279 10.1056/NEJMoa1704795

[30] Heersche N, Lanser DAC, Muntinghe-Wagenaar MB, Mohmaed Ali MI, Ulas EB, Trooster TMA, de Jonge E, Oomen-de Hoop E, Paats MS, Bahce I, et al. Sex and common germline variants affect the toxicity profile and pharmacokinetics of alectinib: a nationwide cohort study in patients with ALK-positive NSCLC. J Thorac Oncol. 2025;20(4):475–486. 39617342 10.1016/j.jtho.2024.11.025

[31] Larkins E, Blumenthal GM, Chen H, He K, Agarwal R, Gieser G, Stephens O, Zahalka E, Ringgold K, Helms W, et al. FDA approval: alectinib for the treatment of metastatic, ALK-positive non-small cell lung cancer following crizotinib. Clin Cancer Res. 2016;22(21):5171–5176. 27413075 10.1158/1078-0432.CCR-16-1293

[32] Dziadziuszko R, Peters S, Ruf T, Cardona A, Guerini E, Kurtsikidze N, Smoljanovic V, Planchard D. Clinical experience and management of adverse events in patients with advanced ALK-positive non-small-cell lung cancer receiving alectinib. ESMO Open. 2022;7(6):100612. 36375271 10.1016/j.esmoop.2022.100612 PMC9663323

[33] Xu X. Alectinib preparation method. Suzhou Miracpharma Technology Co., Ltd.; 2015. CN104402862A.

[34] Keating GM. Elbasvir/grazoprevir: first global approval. Drugs. 2016;76(5):617–624. 26943930 10.1007/s40265-016-0558-3

[35] Bell AM, Wagner JL, Barber KE, Stover KR. Elbasvir/grazoprevir: a review of the latest agent in the fight against hepatitis C. Int J Hepatol. 2016;2016:3852126–3852128. 27403342 10.1155/2016/3852126 PMC4925941

[36] Gane EJ, Pianko S, Roberts SK, Thompson AJ, Zeuzem S, Zuckerman E, Ben-Ari Z, Foster GR, Agarwal K, Laursen AL, et al. Safety and efficacy of an 8-week regimen of grazoprevir plus ruzasvir plus uprifosbuvir compared with grazoprevir plus elbasvir plus uprifosbuvir in participants without cirrhosis infected with hepatitis C virus genotypes 1, 2, or 3 (C-CREST-1 and C-CREST-2, part A): two randomised, phase 2, open-label trials. Lancet Gastroenterol Hepatol. 2017;2(11):805–813. 28802816 10.1016/S2468-1253(17)30159-0

[37] Mangion I, Chen C-Y, Jeon I, Chen Y, Li H, Nguyen HN, Maligres PE, Klapars A, Zavialov I, Yasuda N. Process for preparing HCV NS5A inhibitor tetracyclic heterocycle compounds end-capped with peptides. Merck Sharp & Dohme Corp.; 2015. WO2015065821A1.

[38] Syed YY. Rucaparib: first global approval. Drugs. 2017;77(5):585–592. 28247266 10.1007/s40265-017-0716-2

[39] Liao M, Beltman J, Giordano H, Harding TC, Maloney L, Simmons AD, Xiao JJ. Clinical pharmacokinetics and pharmacodynamics of rucaparib. Clin Pharmacokinet. 2022;61(11):1477–1493. 36107395 10.1007/s40262-022-01157-8 PMC9652254

[40] Raedler LA. Rubraca (Rucaparib) second PARP inhibitor approved for patients with advanced, BRCA-positive ovarian cancer. 2017. p. 2164–1153.

[41] Monk BJ, Parkinson C, Lim MC, O’Malley DM, Oaknin A, Wilson MK, Coleman RL, Lorusso D, Bessette P, Ghamande S, et al. A randomized, phase III trial to evaluate rucaparib monotherapy as maintenance treatment in patients with newly diagnosed ovarian cancer. J Clin Oncol. 2022;40(34):3952–3964. 35658487 10.1200/JCO.22.01003 PMC9746782

[42] Tookman L, Krell J, Nkolobe B, Burley L, McNeish IA. Practical guidance for the management of side effects during rucaparib therapy in a multidisciplinary UK setting. Ther Adv Med Oncol. 2020;12:1758835920921980. 32523631 10.1177/1758835920921980 PMC7257860

[43] Gillmore AT, Badland M, Crook CL, Castro NM, Critcher DJ, Fussell SJ, Jones KJ, Jones MC, Kougoulos E, Mathew JS, et al. Multkilogram scale-up of a reductive alkylation route to a novel PARP inhibitor. Org Process Res Dev. 2012;16(12):1897–1904.

[44] Agrawal V, Garcia JM. The macimorelin-stimulated growth hormone test for adult growth hormone deficiency diagnosis. Expert Rev Mol Diagn. 2014;14(6):647–654. 24834478 10.1586/14737159.2014.915746

[45] Garcia JM, Biller BMK, Korbonits M, Popovic V, Luger A, Strasburger CJ, Chanson P, Medic-Stojanoska M, Schopohl J, Zakrzewska A, et al. Macimorelin as a diagnostic test for adult GH deficiency. J Clin Endocrinol Metab. 2018;103(8):3083–3093. 29860473 10.1210/jc.2018-00665

[46] Guerlavais V, Boeglin D, Mousseaux D, Oiry C, Heitz A, Deghenghi R, Locatelli V, Torsello A, Ghé C, Catapano F, et al. New active series of growth hormone secretagogues. J Med Chem. 2003;46(7):1191–1203. 12646029 10.1021/jm020985q

[47] Hoy SM. Elexacaftor/ivacaftor/tezacaftor: first approval. Drugs. 2019;79(18):2001–2007. 31784874 10.1007/s40265-019-01233-7

[48] Uluer AZ, MacGregor G, Azevedo P, Indihar V, Keating C, Mall MA, McKone EF, Ramsey BW, Rowe SM, Rubenstein RC, et al. Safety and efficacy of vanzacaftor-tezacaftor-deutivacaftor in adults with cystic fibrosis: randomised, double-blind, controlled, phase 2 trials. Lancet Respir Med. 2023;11(6):550–562. 36842446 10.1016/S2213-2600(22)00504-5 PMC12815409

[49] Hadida-Ruah SS, Grootenhuis PDJ, Van Goor FF, Zhou J, Bear BR, Miller MT, McCartney J, Numa MMD, Yang X, Nair N. Preparation of acylaminoindole compounds as modulators of ATP-binding cassette transporters. Vertex Pharmaceuticals Incorporated; 2016. US2009131492A1.

[50] Dhillon S, Keam SJ. Bremelanotide: first approval. Drugs. 2019;79(14):1599–1606. 31429064 10.1007/s40265-019-01187-w

[51] Edinoff AN, Sanders NM, Lewis KB, Apgar TL, Cornett EM, Kaye AM, Kaye AD. Bremelanotide for treatment of female hypoactive sexual desire. Neurol Int. 2022;14(1):75–88. 35076581 10.3390/neurolint14010006 PMC8788464

[52] Kingsberg SA, Clayton AH, Portman D, Williams LA, Krop J, Jordan R, Lucas J, Simon JA. Bremelanotide for the treatment of hypoactive sexual desire disorder: two randomized phase 3 trials. Obstet Gynecol. 2019;134(5):899–908. 31599840 10.1097/AOG.0000000000003500 PMC6819021

[53] Flora D, Mo H, Mayer JP, Khan MA, Yan LZ. Detection and control of aspartimide formation in the synthesis of cyclic peptides. Bioorg Med Chem Lett. 2005;15(4):1065–1068. 15686913 10.1016/j.bmcl.2004.12.025

[54] Jie C, Treyer V, Schibli R, Mu L. Tauvid™: the first FDA-approved PET tracer for imaging tau pathology in Alzheimer’s disease. Pharmaceuticals. 2021;14(2):110. 33573211 10.3390/ph14020110 PMC7911942

[55] Lu M, Pontecorvo MJ, Devous MD Sr., Arora AK, Galante N, McGeehan A, Devadanam C, Salloway SP, Doraiswamy PM, Curtis C, et al. Aggregated tau measured by visual interpretation of flortaucipir positron emission tomography and the associated risk of clinical progression of mild cognitive impairment and Alzheimer disease: results from 2 phase III clinical trials. JAMA Neurol. 2021;78(4):445–453. 33587110 10.1001/jamaneurol.2020.5505 PMC7885097

[56] Fleisher AS, Pontecorvo MJ, Devous MD Sr., Lu M, Arora AK, Truocchio SP, Aldea P, Flitter M, Locascio T, Devine M, et al. Positron emission tomography imaging with [18F]flortaucipir and postmortem assessment of Alzheimer disease neuropathologic changes. JAMA Neurol. 2020;77(7):829–839. 32338734 10.1001/jamaneurol.2020.0528 PMC7186920

[57] Gao M, Wang M, Zheng QH. Fully automated synthesis of [(18)F]T807, a PET tau tracer for Alzheimer’s disease. Bioorg Med Chem Lett. 2015;25(15):2953–2957. 26048805 10.1016/j.bmcl.2015.05.035

[58] Markham A. Lurbinectedin: first approval. Drugs. 2020;80(13):1345–1353. 32816202 10.1007/s40265-020-01374-0

[59] Anobile DP, Bironzo P, Picca F, Lingua MF, Morena D, Righi L, Napoli F, Papotti MG, Pittaro A, Di Nicolantonio F, et al. Evaluation of the preclinical efficacy of lurbinectedin in malignant pleural mesothelioma. Cancers. 2021;13(10):2332. 34066159 10.3390/cancers13102332 PMC8151304

[60] Flores M, Francesch A, Gallego P, Chicharro JL, Zarzuelo M, Fernandez C, Manzanares I. Preparation of antitumoral ecteinascidin derivatives. Pharma Mar, S.A. Ruffles, Graham Keith; 2001. WO2001087894A1.

[61] Markham A. Setmelanotide: first approval. Drugs. 2021;81(3):397–403. 33638809 10.1007/s40265-021-01470-9

[62] Haqq AM, Chung WK, Dollfus H, Haws RM, Martos-Moreno G, Poitou C, Yanovski JA, Mittleman RS, Yuan G, Forsythe E, et al. Efficacy and safety of setmelanotide, a melanocortin-4 receptor agonist, in patients with Bardet-Biedl syndrome and Alström syndrome: a multicentre, randomised, double-blind, placebo-controlled, phase 3 trial with an open-label period. Lancet Diabetes Endocrinol. 2022;10(12):859–868. 36356613 10.1016/S2213-8587(22)00277-7 PMC9847480

[63] Dong ZX. Process for the synthesis of melanocortin analog Ac-Arg-cyclo(Cys-D-Ala-His-D-Phe-Arg-Trp-Cys)-NH_2_. Ipsen Pharma S.A.S.; 2011. WO2011060355A1.

[64] Markham A. Mobocertinib: first approval. Drugs. 2021;81(17):2069–2074. 34716908 10.1007/s40265-021-01632-9

[65] Vasconcelos P, Kobayashi IS, Kobayashi SS, Costa DB. Preclinical characterization of mobocertinib highlights the putative therapeutic window of this novel EGFR inhibitor to EGFR exon 20 insertion mutations. JTO Clin Res Rep. 2021;2(3):100105. 33728415 10.1016/j.jtocrr.2020.100105 PMC7959160

[66] Gonzalvez F, Vincent S, Baker TE, Gould AE, Li S, Wardwell SD, Nadworny S, Ning Y, Zhang S, Huang W-S, et al. Mobocertinib (TAK-788): a targeted inhibitor of EGFR Exon 20 insertion mutants in non-small cell lung cancer. Cancer Discov. 2021;11(7):1672–1687. 33632773 10.1158/2159-8290.CD-20-1683

[67] Green preparation of mobocertinib drug for lung cancer. Shanghai Tin Tsz Bio Valley Biological Engineering Co., Ltd.; 2023. CN116987065A.

[68] Shirley M. Etrasimod: first approval. Drugs. 2024;84(2):247–254. 38388871 10.1007/s40265-024-01997-7

[69] Al-Shamma H, Lehmann-Bruinsma K, Carroll C, Solomon M, Komori HK, Peyrin-Biroulet L, Adams J. The selective sphingosine 1-phosphate receptor modulator etrasimod regulates lymphocyte trafficking and alleviates experimental colitis. J Pharmacol Exp Ther. 2019;369(3):311–317. 30872391 10.1124/jpet.118.254268

[70] Buzard DJ, Kim SH, Lopez L, Kawasaki A, Zhu X, Moody J, Thoresen L, Calderon I, Ullman B, Han S, et al. Discovery of APD334: design of a clinical stage functional antagonist of the sphingosine-1-phosphate-1 receptor. ACS Med Chem Lett. 2014;5(12):1313–1317. 25516790 10.1021/ml500389m PMC4265817

[71] Sengupta D, Gharbaoui T, Krishnan A, Buzard DJ, Jones RM, Ma Y-A, Burda R, Garrido Montalban A, Semple G. An efficient scale-up process for the preparation of the APD334 precursor 4-chloromethyl-1-cyclopentyl-2-(trifluoromethyl)benzene. Org Process Res Dev. 2015;19(6):618–623.

[72] Montalban AG, Buzard DJ, Demattei JA, Gharbaoui T, Johannsen SR, Krishnan AM, Khulman YM, Ma Y-A, Martinelli MJ, Sato SM, et al. Processes for the preparation of (r)-2-(7-(4-cyclopentyl-3-(trifluoromethyl)benzyloxy)-1,2,3,4-tetrahydrocyclopenta[b]indol-3-yl)acetic acid and salts thereof. Arena Pharmaceuticals, Inc.; 2014. US8853419B2.

[73] Syed YY. Iptacopan: first approval. Drugs. 2024;84(5):599–606. 38517653 10.1007/s40265-024-02009-4

[74] Mainolfi N, Ehara T, Karki RG, Anderson K, Mac Sweeney A, Liao SM, Argikar UA, Jendza K, Zhang C, Powers J, et al. Discovery of 4-((2S,4S)-4-ethoxy-1-((5-methoxy-7-methyl-1H-indol-4-yl)methyl)piperidin-2-yl)benzoic Acid (LNP023), a factor B inhibitor specifically designed to be applicable to treating a diverse array of complement mediated diseases. J Med Chem. 2020;63(11):5697–5722. 32073845 10.1021/acs.jmedchem.9b01870

[75] Castellino NJ, Montgomery AP, Danon JJ, Kassiou M. Late-stage functionalization for improving drug-like molecular properties. Chem Rev. 2023;123(13):8127–8153. 37285604 10.1021/acs.chemrev.2c00797

[76] Guillemard L, Ackermann L, Johansson MJ. Late-stage meta-C–H alkylation of pharmaceuticals to modulate biological properties and expedite molecular optimisation in a single step. Nat Commun. 2024;15(1):3349. 38637496 10.1038/s41467-024-46697-8 PMC11026381

[77] Shu YZ, Johnson BM, Yang TJ. Role of biotransformation studies in minimizing metabolism-related liabilities in drug discovery. AAPS J. 2008;10(1):178–192. 18446518 10.1208/s12248-008-9016-9 PMC2751461

[78] Lazzara PR, Moore TW. Scaffold-hopping as a strategy to address metabolic liabilities of aromatic compounds. RSC Med Chem. 2020;11(1):18–29. 33479602 10.1039/c9md00396g PMC7451012

[79] Sun H, Scott DO. Structure-based drug metabolism predictions for drug design. Chem Biol Drug Des. 2010;75(1):3–17. 19878193 10.1111/j.1747-0285.2009.00899.x

[80] Babalola BA, Malik M, Olowokere O, Adebesin A, Sharma L. Indoles in drug design and medicinal chemistry. Eur J Med Chem Rep. 2025;13:100252.

[81] Zhang X, Lu X, Zhang P, Dai M, Liang T. Recent advances in the multicomponent reactions of indoles. Eur J Org Chem. 2025;28(15):e202401446.

[82] Bawazir WA, Ain Q. Indole-imidazole hybrids as emerging therapeutic scaffolds: synthetic advances and biomedical applications. Molecules. 2025;30(21):4164. 41226127 10.3390/molecules30214164 PMC12608492

[83] N MK, Nargund S, Murugan V, Gote A. Indole-based scaffolds in medicinal chemistry: recent advances and perspectives. Int J Res Publ Rev. 2025;6(8):5975–5983.

